# Adenosine A1 receptor ligands bind to α-synuclein: implications for α-synuclein misfolding and α-synucleinopathy in Parkinson’s disease

**DOI:** 10.1186/s40035-022-00284-3

**Published:** 2022-02-10

**Authors:** Elisabet Jakova, Mohamed Taha Moutaoufik, Jeremy S. Lee, Mohan Babu, Francisco S. Cayabyab

**Affiliations:** 1 Department of Surgery, College of Medicine, University of Saskatchewan, Saskatoon, SK Canada; 2 Department of Chemistry and Biochemistry, Faculty of Science, University of Regina, Regina, SK Canada; 3 Department of Biochemistry, Microbiology and Immunology, College of Medicine, University of Saskatchewan, Saskatoon, SK Canada

**Keywords:** Alpha-synucleinopathy, Adenosine A1 receptor, N^6^-cyclopentyladenosine, 8-cyclopentyl-1,3-dipropylxanthine, 1-aminoindan, 2-aminoindan, Neuroprotection, Neurodegeneration, Protein misfolding

## Abstract

**Background:**

Accumulating α-synuclein (α-syn) aggregates in neurons and glial cells are the staples of many synucleinopathy disorders, such as Parkinson’s disease (PD). Since brain adenosine becomes greatly elevated in ageing brains and chronic adenosine A1 receptor (A1R) stimulation leads to neurodegeneration, we determined whether adenosine or A1R receptor ligands mimic the action of known compounds that promote α-syn aggregation (e.g., the amphetamine analogue 2-aminoindan) or inhibit α-syn aggregation (e.g., Rasagiline metabolite 1-aminoindan). In the present study, we determined whether adenosine, A1R receptor agonist N^6^-Cyclopentyladenosine (CPA) and antagonist 8-cyclopentyl-1,3-dipropylxanthine (DPCPX) could directly interact with α-syn to modulate α-syn aggregation and neurodegeneration of dopaminergic neurons in the substantia nigra (SN).

**Methods:**

Nanopore analysis and molecular docking were used to test the binding properties of CPA and DPCPX with α-syn in vitro. Sprague–Dawley rats were administered with 7-day intraperitoneal injections of the A1R ligands and 1- and 2-aminoindan, and levels of α-syn aggregation and neurodegeneration were examined in the SN pars compacta and hippocampal regions using confocal imaging and Western blotting.

**Results:**

Using nanopore analysis, we showed that the A1R agonists (CPA and adenosine) interacted with the N-terminus of α-syn, similar to 2-aminoindan, which is expected to promote a “knot” conformation and α-syn misfolding. In contrast, the A1R antagonist DPCPX interacted with the N- and C-termini of α-syn, similar to 1-aminoindan, which is expected to promote a “loop” conformation that prevents α-syn misfolding. Molecular docking studies revealed that adenosine, CPA and 2-aminoindan interacted with the hydrophobic core of α-syn N-terminus, whereas DPCPX and 1-aminoindan showed direct binding to the N- and C-terminal hydrophobic pockets. Confocal imaging and Western blot analyses revealed that chronic treatments with CPA alone or in combination with 2-aminoindan increased α-syn expression/aggregation and neurodegeneration in both SN pars compacta and hippocampus. In contrast, DPCPX and 1-aminoindan attenuated the CPA-induced α-syn expression/aggregation and neurodegeneration in SN and hippocampus.

**Conclusions:**

The results indicate that A1R agonists and drugs promoting a “knot” conformation of α-syn can cause α-synucleinopathy and increase neuronal degeneration, whereas A1R antagonists and drugs promoting a “loop” conformation of α-syn can be harnessed for possible neuroprotective therapies to decrease α-synucleinopathy in PD.

**Supplementary Information:**

The online version contains supplementary material available at 10.1186/s40035-022-00284-3.

## Background

Adenosine is a nucleoside that is involved in many physiological activities including cell proliferation, migration of dendritic cells, and the release of small proteins called cytokines which are vital for cell signalling from periphery to secondary lymphoid organs, vascular reactivity, apoptosis and most importantly, the passage of neuronal stem cells [1–5]. Adenosine is also implicated in central nervous system (CNS) disorders such as ischemia, trauma, epilepsy, neuropsychiatric disorders and cancer [6–11]. Moreover, various roles of adenosine have garnered intense investigations in many ageing-related neurodegenerative diseases such as ischemic stroke, Alzheimer’s disease (AD) and Parkinson’s disease (PD) [12–15]. PD is the second most prevalent ageing-related neurodegenerative disease after AD [16]. The pathophysiology of PD directly involves the imbalance of dopaminergic signalling pathways and accumulation of protein aggregates of α-synuclein (α-syn) in inclusions (Lewy bodies) causing the characteristic motor and cognitive deficits commonly observed in PD patients [17–19]. Recently, some neuroprotective drugs have been found to bind to α-syn and prevent further aggregation, including caffeine, nicotine, 1-aminoindan and metformin [20]. Additionally, there are other drugs such as methamphetamine, cocaine, 2-aminoindan and the herbicides, paraquat and rotenone, which appear to be neurotoxic because they increase α-syn misfolding and can be correlated with a higher incidence of PD [20–23]. Chronic adenosine A1 receptor (A1R) stimulation has recently been reported to cause hippocampal and substantia nigra (SN) neuronal death, as well as increasing α-syn accumulation in dopaminergic SN neurons [24, 25]. Since a primary therapeutic goal of management of PD is to minimize α-syn misfolding and aggregation, we investigated whether adenosine and the A1R agonist N^6^-cyclopentyladenosine (CPA), as well as antagonist 8-cyclopentyl-1,3-dipropylxanthine (DPCPX), can bind to and modulate α-syn misfolding.

A1Rs are expressed at high levels in the limbic system especially in the hippocampus as well as in the SN region. A1R stimulation with CPA reduces glutamate and gamma-aminobutyric acid release from nerve terminals of the SN pars reticulata region of the rat brain [26]. This presynaptic inhibition of glutamate release from the subthalamonigral pathway may be clinically relevant in improving tardive dyskinesia in PD patients by reducing the glutamatergic outputs of the SN pars reticulata dopaminergic neurons. Moreover, most compounds that target A1R activation were believed to be neuroprotective in both the SN and hippocampus. For example, activation of A1R is involved in paeoniflorin (a chemical compound derived from *Paenoia lactiflora*)-induced neuroprotection in cerebral ischemia in Sprague–Dawley rats [27]. However, we recently reported that chronic stimulation of A1Rs by intraperitoneal (i.p.) injection of the A1R agonist CPA in rats for 3 days is sufficient to induce neurodegeneration in the hippocampus [24]. Additionally, longer-term chronic A1R stimulation with CPA increases sortilin expression that promotes α-syn upregulation in dopaminergic MN9D cells and SN dopaminergic neurons of Sprague–Dawley rats [25]. Since highly upregulated α-syn can be found in the hippocampus and SN of rodent synucleinopathy models [25, 28–30], we therefore tested the possibility that the commonly used A1R-selective agonist ligand CPA can bind to α-syn and enhance neurotoxicity, whereas the A1R-specific antagonist ligand DPCPX can bind to α-syn and promote neuroprotection. By 7-day chronic injections in Sprague–Dawley male rats, we determined if chronic stimulation with CPA causes dopaminergic neuron loss and increases expression of α-syn in the SN. We then co-administered DPCPX as a method to control neurodegeneration and decrease aggregation of α-syn caused initially by CPA. Fluoro-Jade C (FJC) and Thioflavin S (Thio-S) staining of hippocampal and SN brain regions were performed to assess neurodegeneration and α-syn aggregation, respectively [31, 32].

**Fig. 1:**
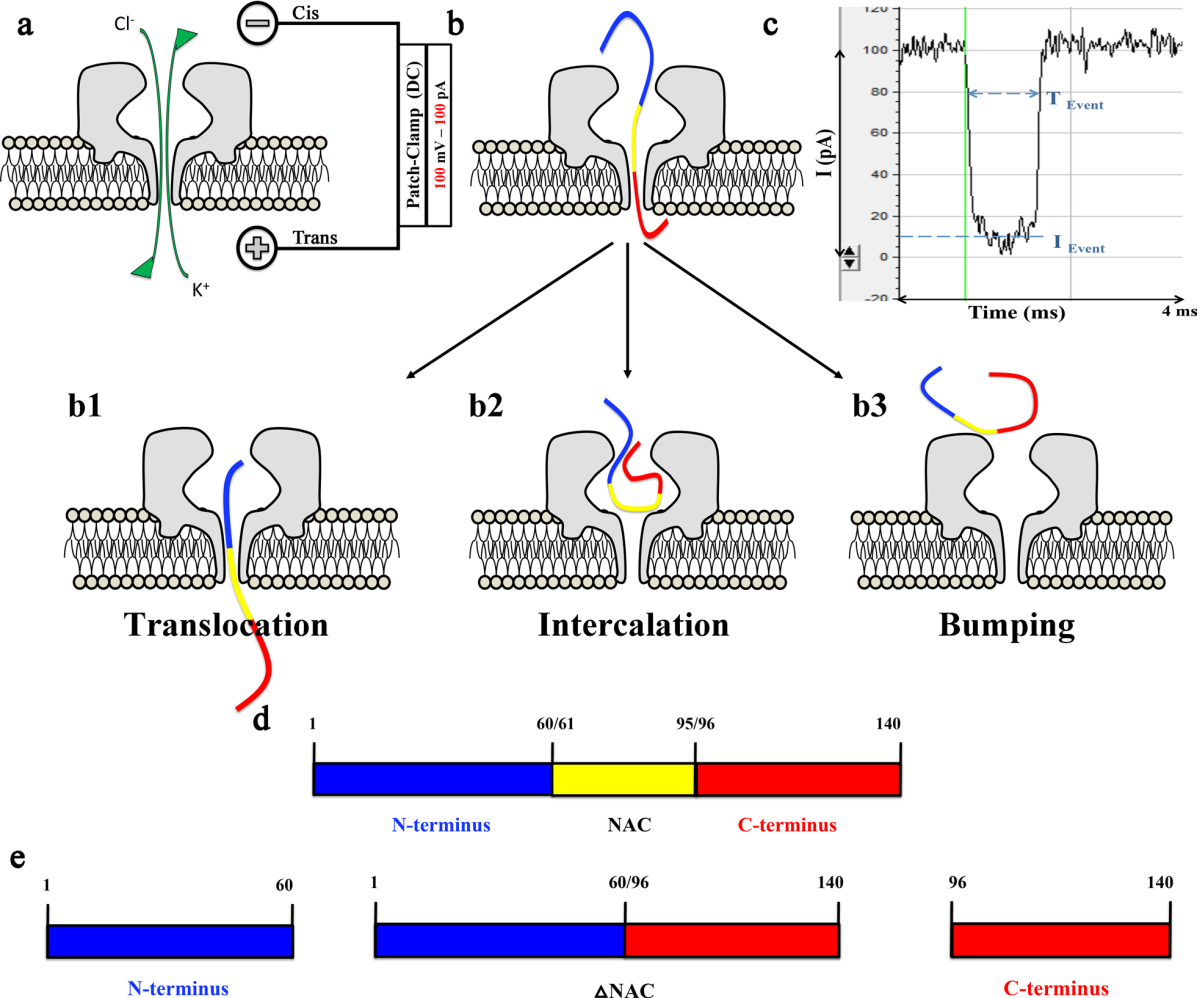
Nanopore analysis setup and α-synuclein (α-syn) interaction with the α-Hemolysin pore. **a** The patch-clamp setup at 100 mV direct current (DC) allows the ions to flow in the pore and create an ionic current **b** The interruption of the current when α-syn interacts with the pore forming three distinguishable blockade current events: **b1** Translocation events, where α-syn goes through the pore causing a large current blockade (as seen in Fig. 1** c**); **b2** Intercalation events, where α-syn is trapped in the pore entrance, but will diffuse back after a period of time causing an intermediate current blockade; **b3** Bumping events, where α-syn approaches the pore, but diffuses away without entering causing a small current blockade. **c** Disruption of the blockade current and time caused by α-syn when the protein translocates the pore. **d** Full sequence of α-syn. **e** The domains of α-syn used in the nanopore setup consisting of: N-terminus (blue); ΔNAC, the entire sequence of α-synuclein without the non-amyloid β-component region (blue and red); and C-terminus (red)

Nanopore analysis and molecular docking are useful analytical tools for studying intrinsically disordered proteins like cellular prion proteins, β-amyloid as well as α-syn and α-syn/drug complexes [20, 21, 33–39]. Nanopores are single-molecule counters consisting of a nanometre aperture that allows the fluxes of ions and small charged polypeptides through an insulating membrane. Applying a voltage across this membrane results in an electrochemical gradient that drives ions through the α-hemolysin (α-HML) toxin derived from *Staphylococcus aureus* [33]. A single α-syn protein interacts with the α-HML pore, causing a blockade current (*I*) for an amount of time (*T*) (Fig. 1) [39]. When α-syn translocates through the pore, a large current blockade is observed for a long translocation time. Conversely, if the α-syn protein approaches the pore but then diffuses away without entering, a small current blockade is observed for a short time. This type of event is called bumping [40, 41]. The most important advantage of nanopore analysis is that molecules can be detected without labelling and at very low concentrations. Uniquely, this technique requires less than an hour to non-destructively analyze thousands of single molecules. Moreover, this technique has been widely used to determine if a drug binds to α-syn. When a protein-drug complex is formed, there is an increase of bumping events and a decrease in translocation events, which indicates that the drug causes folding of the protein.

Additionally, molecular docking is a computer simulation technique that allows prediction of the binding conformation of a desired protein or peptide to a chemical compound or other small molecules, making molecular docking analysis one of the best techniques for structure-based drug design [42, 43]. Therefore, using these complimentary biophysical and computational techniques in combination with our in vivo studies, we aimed to elucidate the effects of adenosine and other A1R ligands on α-syn conformations and dopaminergic neuron loss in PD.

## Methods

### Animal protocol

Animals were housed and treated humanely in accordance with the guidelines from the following governing bodies: National Research Council (US) Committee for the Update of the Guide for the Care and Use of Laboratory Animals (Washington DC, 2011); Canadian Council on Animal Care (CCAC); and the University of Saskatchewan Animal Research Ethics Board (AREB) that approved our Animal Use Protocol (#20070090). Male Sprague–Dawley rats (20–30 days old varying from 250 to 300 g) were used for immunofluorescence confocal imaging and biochemical studies as described below. The animals were housed in cages of two, with free access to food pellets and water.

### Reagents

α-Syn (rPeptide, Bogart, GA) was dissolved in nuclease-free water at a final concentration of 1 μM. Adenosine was purchased from Millipore-Sigma (Oakville, Canada), CPA was purchased from Abcam (Toronto, Canada), DPCPX from Tocris (Burlington, Canada), 1- and 2-aminoindan were purchased from Sigma-Aldrich (Oakville, Canada). For the nanopore analysis, all drugs were dissolved in methanol (MeOH) and used at a final concentration of 10 μM. For the 7-day chronic i.p. injection, CPA, DPCPX, 1- and 2-aminoindan were dissolved in 0.1% dimethyl sulfoxide (DMSO) (Sigma-Aldrich, Oakville, Canada) in 0.9% sodium chloride at a final concentration of 3 mg/ml.

### Nanopore analysis

#### Instrument setup

The standard direct current (DC) setup has been described in detail previously by our lab [39, 44, 45]. In brief, a lipid bilayer was painted onto a 150-μm aperture in a Teflon perfusion cup. The two buffer compartments on either side of the lipid bilayer each contained a 1-ml total volume. Five microliters of 1 μg/ml α-HML (Millipore-Sigma, Oakville, Canada) were added to the *cis* side of the membrane and the current was monitored until stable pore insertion was achieved. Consistent results were achieved with one to four pores. The peptides were added to the *cis* side of the pore with a positive electrode on the *trans*-side. The experiments were carried out at 22 ± 1 °C with an applied potential of 100 mV at a bandwidth of 10 kHz using an Axopatch 200B amplifier (Axon Instruments, San Jose, CA) under voltage-clamp conditions using a Clampfit software (Molecular Devices, San Jose, CA). As discussed elsewhere, temperature changes due to Joule heating are expected to be negligible [46].

#### Data analysis

The blockade amplitudes and duration times obtained with Clampfit were transferred to Origin 7 graphing software (OriginLab Corporation, Northampton, MA) and were used to construct blockade current and time histograms. The blockade amplitudes were plotted as statistical histograms and each event population (e.g., translocation, intercalation and bumping) was fitted with a Gaussian function to obtain the peak/population blockade current value (*I*). The duration time data for each population were plotted separately and the data fitted with a single exponential decay function to obtain the characteristic time (*T*). Each experiment was repeated at least three times and the event profiles were added together. The error in the peak current was estimated to be <  ± 1 pA and the proportion (%) of the events in each peak is reported in Tables as means ± SEM.

### Structural modeling and docking

Distinct structural subpopulations of α-syn monomers (C1–C8, see Additional file 1: Appendix Fig. 1) have recently been identified [47] and were subsequently used in the present study (with the exception of C6, which is believed to be membrane-bound) in our molecular docking simulations to predict the drug-protein complexes. The respective conformation of each of the α-syn structures was taken from PDB-DEV (Entry: PDBDEV_00000082) [48]. The chemical structures of adenosine, CPA, DPCPX, 1-aminoindan and 2-aminoindan were obtained from PubChem (CIDs: 60961, 53477947, 1329, 123445, 76310, respectively). The molecular docking study was carried out using Autodock Vina module implemented in PyRx tool (La Jolla, CA) [43]. Protein and ligand interactions were analyzed and visualized through Pymol (New York, NY) and LigPlot + (Cambridge, UK).

### In vivo drug treatments to study α-syn aggregation and neurodegeneration

In support of the in vitro data, a full in vivo study consisting of 7-day chronic i.p. injections of eight reagents or their combinations in 28-day old male Sprague–Dawley rats was performed. The eight treatments consisted of (1) Control (0.1% DMSO in 0.9% saline), (2) CPA, (3) DPCPX, (4) 1-aminoindan, (5) 2-aminoindan, (6) CPA + DPCPX, (7) 1-aminoindan + CPA, and (8) 2-aminoindan + CPA. Although 1-aminoindan and 2-aminoindan have very similar structures, they have been shown to possess very different properties in vitro [21, 36], therefore we suggest they will exhibit different physiological properties in vivo as well. All drugs were dissolved at 3 mg/ml in DMSO, and each drug was administered to the animals by daily i.p. injections (3 mg/kg body weight) for 7 consecutive days. After the first injection with DPCPX, 1-aminoindan or 2-aminoindan, the animals were returned to their cages for 30 min before a subsequent CPA injection was administered. Then on the eighth day following the final injections, the animals were sacrificed and processed for brain immunohistochemistry/confocal imaging or Western blotting as described below.

### Immunohistochemistry

Anesthetized rats were transcardially perfused with 0.9% saline, and then fixed with 4% paraformaldehyde. The extracted brains were put in 30% cryoprotected sucrose solution for 48 h prior to slicing. The brains were initially frozen at − 40 °C (BFS-30 mp controllers) and sliced with the help of a microtome (Leica SM2010 R Sliding controller). Coronal slices of 40 μm were then washed three times in 0.1 M phosphate buffered saline followed by 1-h blocking at room temperature with blocking buffer. The buffer solution components have been previously described [49]. The slices were then incubated overnight at 4 °C with the following primary antibodies: 1:200 mouse monoclonal to α-syn (Abcam Inc, Toronto, Canada) and 1:200 rabbit polyclonal to tyrosine hydroxylase (TH) (Millipore-Sigma, Oakville, Canada). Subsequently, slices were then incubated for 1 h in the dark at room temperature with the following secondary antibodies: AlexaFluor-555-conjugated anti-mouse and AlexaFluor-647-conjugated anti-rabbit (1:1000) purchased from Invitrogen (Thermo Fisher Scientific, Waltham, MA). Slices were then treated with Thio-S (see further details below). Lastly, the slices were incubated for 5 min at room temperature with DAPI (2 mg/ml) from Invitrogen (Thermo Fisher Scientific, Waltham, MA) and images were taken using a Zeiss LSM700 confocal microscope (Carl Zeiss Group, Canada) and analyzed with ImageJ (Public Domain). Images of the hippocampal CA1 pyramidal layer and the SN pars compacta were obtained using the Zeiss Plan-Apochromat 63X/1.4 oil objective lens (Carl Zeiss). Images were acquired as Z-stack images of hippocampal or SN regions with 12–13 Z-stack images taken at 1-µm intervals near the middle of brain slices. Two Z-stack images were taken along the hippocampal CA1 or SN pars compacta region for each slice, and immunofluorescence signals were averaged using densitometry analysis.

### Thioflavin-S

Thio-S (Sigma-Aldrich, Oakville, Canada) is a fluorescent marker that detects α-syn aggregates and amyloid plaques. Coronal slices of 40 μm were firstly treated with 0.3% KMnO_4_ for 4 min, followed by a 30-min incubation with 1 M phosphate buffered saline at 4 °C. These slices were then stained with 0.05% Thio-S in 50% ethanol in the dark for 8 min, rinsed with 80% ethanol twice, followed by three rinses with ultra-pure water for 30 s. Finally, the slices were incubated again with 1 M phosphate buffered saline for 30 min at 4 °C before starting the DAPI stain. The FITC filter (488 nm laser line) was used to image Thio-S using a Zeiss LSM700 confocal microscope (Carl Zeiss Group, Canada) and images were analyzed with ImageJ (Public Domain).

### Fluoro-Jade C

FJC is a fluorescent marker for neurodegeneration (Millipore-Sigma, Oakville, Canada). Coronal slices of 40 μm were mounted on 5% gelatin-coated super-frost plus microscope slides (Thermo Fisher Scientific, Waltham, MA) and dried overnight at 4 °C. Initially, the microscope slides were immersed in 1% NaOH/80% ethanol for 5 min followed by 2-min immersion in 70% ethanol. The slides were then rinsed for 2 min with ultra-pure water. The microscope slides were further immersed in 0.06% KMnO_4_ for 10 min, followed by additional rinse for 2 min with ultra-pure water. The slides were then stained with 0.004% FJC in 0.1% acetic acid for 20 min with gentle shaking on an orbital shaker. Lastly, the slides were rinsed three times in ultra-pure water for 1 min each, making sure to remove all the excess water after each rinse. The slides were then rinsed in xylene and allowed to dry overnight at 4 °C. Then they were treated with Prolong Gold Antifade Reagent from Invitrogen (Thermo Fisher Scientific, Waltham, MA), and respective images were taken using a Zeiss LSM700 confocal microscope (Carl Zeiss Group, Canada) and analyzed with ImageJ (Public Domain). FJC fluorescence was obtained by exciting the dye with 488 nm laser.

### Nigral slice preparation for Western blotting

Nigral slices (400 μm) from male Sprague–Dawley rats were prepared with the help of the vibratome tissue slicer (Leica VT1200 S). The rat was initially anaesthetized with halothane and rapidly decapitated. Once the brain was extracted it was placed immediately in an ice-cold sucrose dissection medium and oxygenated with 95% oxygen with 5% carbon dioxide. The slices were then equilibrated in the oxygenated artificial cerebrospinal fluid for 1 h. Nigral slices were transferred into homogenization lysis buffer containing 1% NP-40 detergent and supplemented with protease inhibitors. After tissue homogenization, the protein concentration was measured with Bradford Assay using the DC protein assay dye (Bio-Rad, Canada). Protein lysates (50 μg/lane) from the different treatment groups were separated in 12% SDS-PAGE gels, and transferred to polyvinylidene difluoride (PVDF) membranes (Millipore, ThermoFisher Scientific, USA) using 30 V overnight at 4 °C. The PVDF membranes were then treated with mouse monoclonal [4D6] anti-α-syn (Abcam Inc, Canada) primary antibody overnight at 4 °C after 1-h blocking with 5% non-fat milk in Tris-buffered saline with Tween-20. The next day, the membranes were incubated for 1 h with the appropriate secondary antibody at room temperature. The membranes were then finally re-probed with chicken polyclonal antibody against Tubulin-III. Proteins were visualized using enhanced chemiluminescence and ChemiDoc (Bio-Rad, Canada). Densitometry analysis was performed using ImageJ (Public Domain). All the above-mentioned solutions and procedures have been previously described [49].

### Statistical analyses

For nanopore, histological and Western blot analyses, statistical analyses were conducted with GraphPad Prism 8 software (San Diego, CA) with one-way ANOVA followed by Student-Neuman-Keuls multiple comparison post-hoc test. The significances are indicated as: ns, non-significant; *P < 0.05; **P < 0.01; and ***P < 0.001.

## Results

### CPA and DPCPX bind to α-syn in vitro

**Fig. 2:**
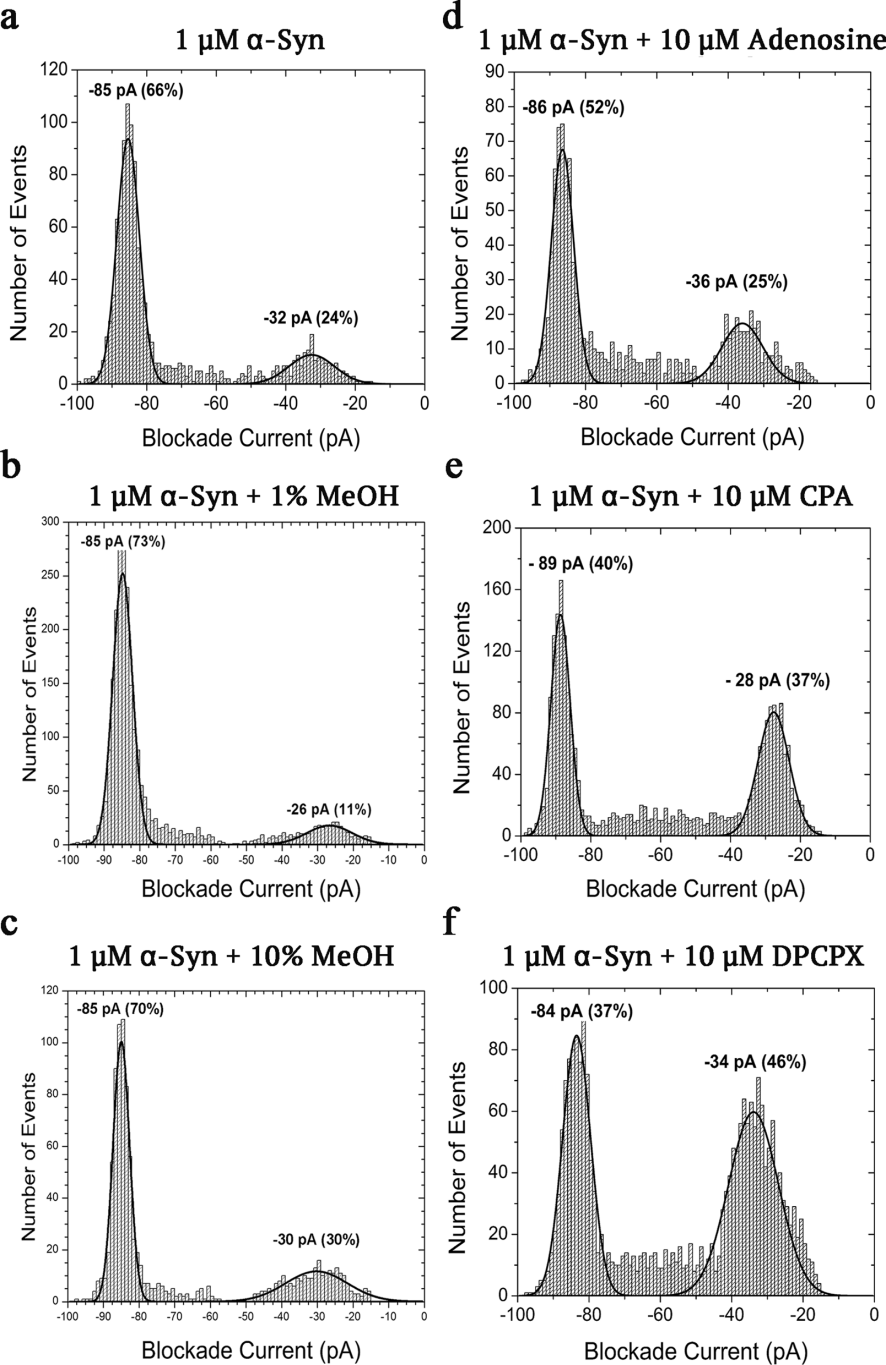
Representative blockade current histograms of 1 μM α-synuclein alone (**a)** and with 1% methanol (**b**), 10% methanol (**c**), 10 μM adenosine (**d**), 10 μM CPA (**e**) and 10 μM DPCPX (**f**) at 100 mV DC, indicating binding to the protein. Each experiment was run in triplicates and the standard error of the mean estimated for the percentage of events was <  ± 10% (see Table 1 and Additional file 1: Table S1)

Initially, both CPA and DPCPX were tested by nanopore analysis. Once a stable pore was created, a final concentration of 1 μM α-syn was inserted on the *cis* side of the perfusion cup. At first, a few bumping events at 30 pA and a higher number of translocation events around 85 pA were observed, whereas intercalation events were rarely encountered (Fig. 1b2). Thus, these observations confirm similar recordings of stable blockade and bumping currents of α-syn, as previously reported [33]. Previous work using confocal single-molecule fluorescence techniques indicated the formation of oligomers of α-syn in the presence of DMSO [50]. These oligomers have a high binding affinity to lipid membranes. Therefore, to avoid potential aggregation of α-syn as well as binding of the oligomers to the membrane and disruption of the lipid bilayer, which could lead to further issues with α-HML assembly and conductivity, we decided to use MeOH to dissolve all the drugs in our nanopore studies. As shown in Fig. 2b and c, 1% and 10% final concentrations of MeOH did not significantly change the blockade current histograms compared to control (α-syn alone, Fig. 2a). The translocation and bumping peaks in the presence of 1% and 10% MeOH were similar to those found in α-syn alone (i.e., blockade current peaks around − 85 pA). Moreover, the blockade populations of the translocation and bumping peaks in the presence of 10% MeOH were not significantly different from the α-syn alone control recordings (Additional file 1: Table S1); therefore, all subsequent recordings with different drugs described below were performed with 10% MeOH in the recording solution.

**Fig. 3:**
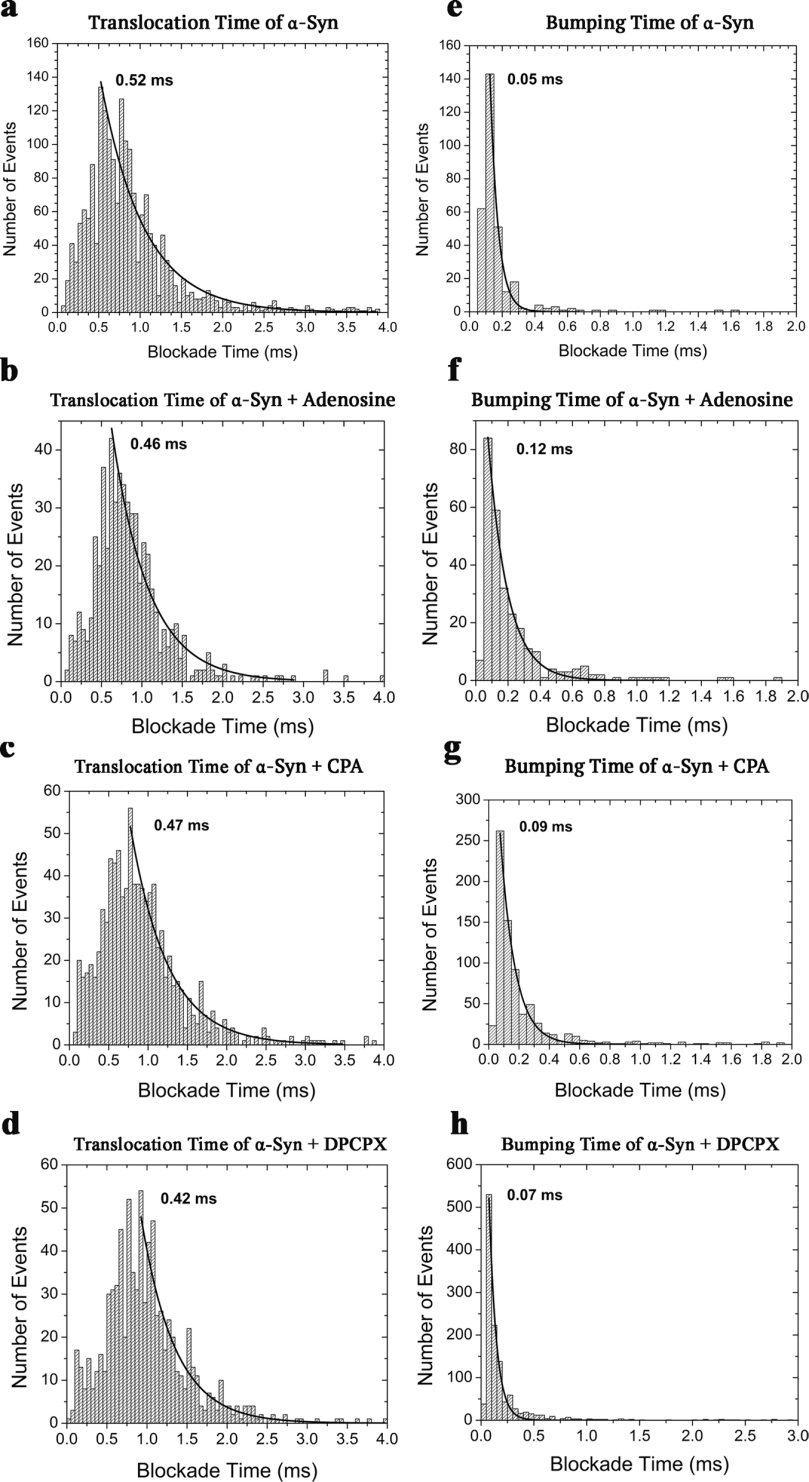
Representative blockade time profiles of translocation (**a**–**d**) and bumping events (**e**–**h**) for 1 μM α-synuclein alone and in the presence of 10 μM adenosine, 10 μM CPA or 10 μM DPCPX. Each experiment was run in triplicates. For mean and SEM values of populations of translocation and bumping and blockade times in the absence or presence of adenosine, CPA or DPCPX, please see Table 1

When 1 µM α-syn and 10 μM of the drug (in 10% MeOH) were inserted on the *cis* side, we observed changes in the blockade current events (See Additional file 1: Fig. S1 for details of current of α-HML pore at + 100 mV and the changes in blockade current events once α-syn and/or drugs such as CPA and DPCPX were added in the *cis* side of the perfusion cup). First, we tested the potential binding of adenosine with α-syn in our nanopore setup. Adenosine appeared to have weak binding affinity to α-syn. The majority of the events of the −86 pA blockade current was related to α-syn translocation; however, there were fewer events observed in the translocation peak in the α-syn and adenosine histogram (52%) compared to α-syn alone (66%) (Fig. 2d). Interestingly, CPA and DPCPX appeared to bind to α-syn as well (Fig. 2e, f). For the first time, we observed a decrease in the blockade current of the translocation peak from −85 pA for α-syn alone to −89 pA for the α-syn and CPA complex, as the percentage of events decreased from 66% to 40% for CPA (Fig. 2e). Taken together, the observed effects of CPA and, to a lesser extent, adenosine on the α-syn translocation events are clear signs of binding. Conversely, DPCPX caused a small increase of the blockade current to -84 pA, which was accompanied by a decrease in the number of events in the translocation peak (Fig. 2f). The blockade times of translocation and bumping peaks of α-syn with and without adenosine, CPA or DPCPX were also calculated. Representative exponential time graphs are shown in Fig. 3. The times of translocation and bumping events for α-syn alone were well established [33]. The times of translocation events (α-syn alone, 0.52 ms) decreased when α-syn was combined with adenosine (0.46 ms), CPA (0.47 ms) or DPCPX (0.42 ms), which further indicates a potential binding of these drugs to the protein. On the other hand, we observed increased bumping times when α-syn was incubated with adenosine, CPA or DPCPX (Fig. 3e–h). A full summary of the blockade populations and times is shown in Table 1.

**Table 1:** Populations and blockade times of translocations and bumping events for α-syn alone and α-syn complexes with adenosine, CPA, and DPCPX

Protein-drug complex	α-syn	α-syn + Adenosine	α-syn + CPA	α-syn + DPCPX
Population of translocation	66%	52% [*]	40% [**]	37% [**]
SEM	1%	2%	1%	1%
Population of bumping	24%	25% [ns]	37% [**]	46% [**]
SEM	1%	3%	2%	2%
Time of translocation	0.52 ms	0.46 ms [ns]	0.47 ms [ns]	0.42 ms [ns]
SEM	0.05 ms	0.05 ms	0.05 ms	0.04 ms
Time of bumping	0.05 ms	0.12 ms [***]	0.09 ms [**]	0.07 ms [*]
SEM	< 0.01 ms	0.01 ms	< 0.01 ms	< 0.01 ms

ns, non-significant

*P < 0.05, **P < 0.01, and ***P < 0.001 *vs* α-syn alone (one-way ANOVA, followed by Student–Newman–Keuls multiple comparison test)

Caffeine, a nonselective inhibitor of all the adenosine receptors (A1, A2A, A2B and A3) [51], has previously been shown by nanopore analysis to bind to the N- and C-termini of α-syn, thereby promoting a neuroprotective loop conformation [37]. It is known that caffeine competitively antagonizes adenosine’s effects [52, 53]. Although the blockade populations of adenosine and CPA showed some similarities to the histogram of caffeine binding to α-syn [37], both adenosine and CPA decreased the blockade current of the translocation events of α-syn to  − 86 and − 89 pA, respectively (Fig. 2d, e). Previous results using 5 μM caffeine showed that the translocation population decreased to 44% from 81% of α-syn alone, whereas the bumping population significantly increased to 38% compared to 9% of α-syn alone [37]. Similarly, here DPCPX + α-syn decreased the translocation population (37%) and increased the bumping population (46%) (Fig. 2f), suggesting that like caffeine, DPCPX may potentially bind to the N- and C-termini of α-syn, forming a loop conformation.

### Alpha-synuclein domain investigations of CPA and DPCPX

**Fig. 4:**
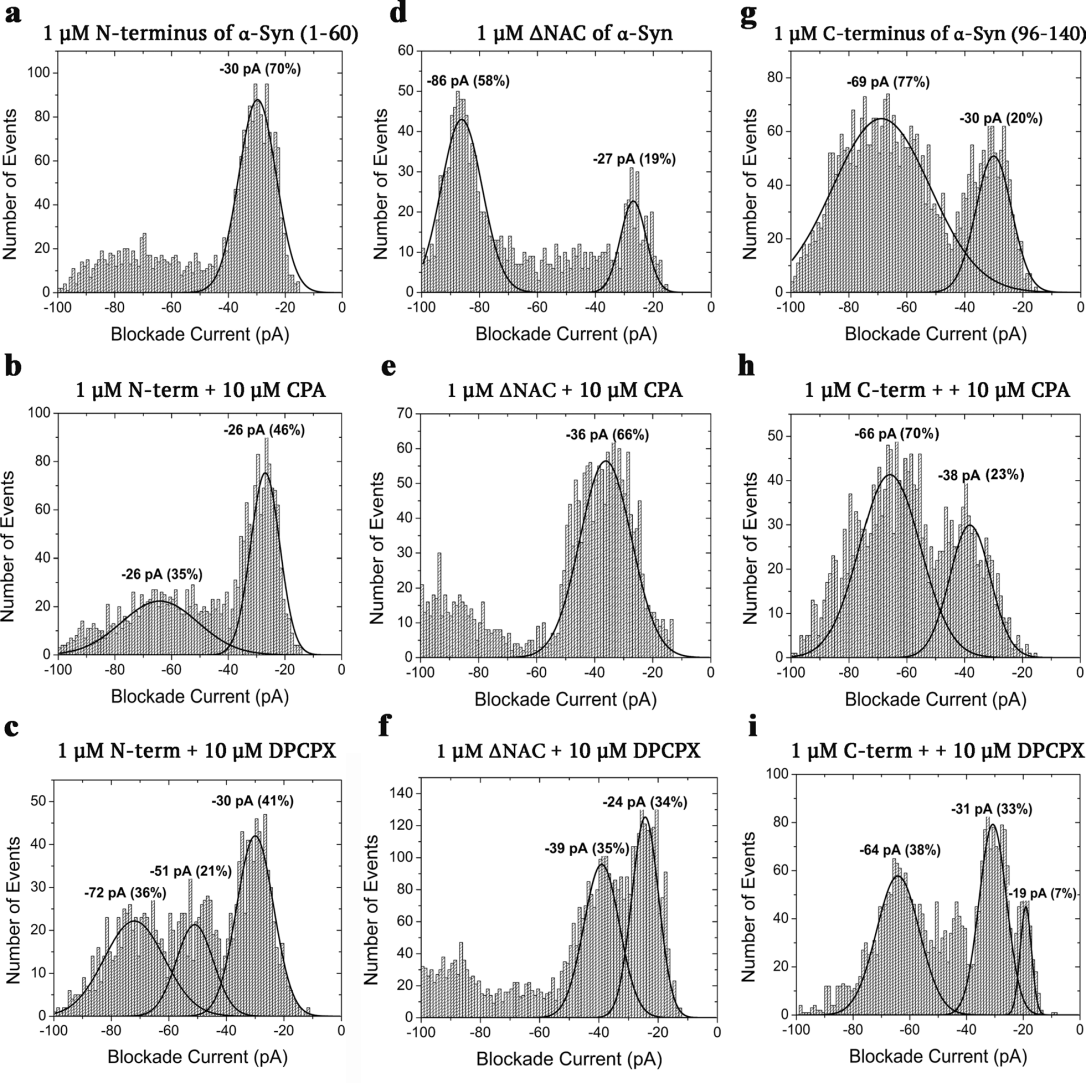
Representative blockade current histograms of 10 μM CPA and 10 μM DPCPX with N-terminus (**a–c**), ΔNAC (**d–f**) and C-terminus (**g–i**) of α-synuclein at 100 mV DC. Each experiment was run in triplicates and the error estimated for the percentage of events was <  ± 10% (see Table 2)

To further probe the exact binding of both CPA and DPCPX, separate domains of α-syn, namely the N- and C-termini, and the ΔNAC construct, i.e., α-syn with deletion of the non-amyloid β-component region (Fig. 1d, e), were tested against CPA or DPCPX. The behaviour of each domain was different in a standard nanopore analysis at a direct current voltage of 100 mV (Fig. 4a, d, g). The blockade current histogram of the N-terminus had a single Gaussian peak at − 30 pA due to bumping events. The N-terminus is positively charged (+ 4). Consequently, it will be difficult for this N-terminal fragment to translocate through the pore under the applied positive transmembrane voltage. Conversely, the C-terminus contains a total of 12 negative charges, which permits translocation through the pore. The blockade current histogram had a large and wide translocation peak at − 69 pA and a fairly small bumping peak at − 30 pA. The ΔNAC had two peaks, a large peak at − 86 pA due to translocation and a smaller one at − 27 pA due to bumping.

Figure 4 shows the blockade current histograms of each α-syn domain in the presence of CPA (Fig. 4b, e, h) or DPCPX (Fig. 4c, f, i). With the addition of CPA, the N-terminus proportion of bumping events decreased significantly from 70% to 46% (Fig. 4a *vs* b). Conversely, the widespread block of events between − 50 and − 100 pA in the N-terminus control developed into a well-defined broad translocation peak at − 26 pA with a population of 35%. The changes observed for ΔNAC after addition of CPA were remarkable and demonstrated clear signs of binding (Fig. 4d *vs* e). The broad translocation peak at − 86 pA was reduced into a small cluster of events, whereas the small bumping peak significantly increased in population from 19% to 66% and shifted to − 36 pA from − 27 pA. Interestingly, the C-terminal domain histogram profiles in the absence and the presence of CPA did not show significant differences (Fig. 4g *vs* h), which indicates that CPA does not interact with the C-terminus.

In contrast, DPCPX produced different profile histograms of each of the α-syn domains when compared to both the control and CPA. The N-terminus histogram profile showed that DPCPX caused a decrease in the proportion of bumping events from 70% to 41%, and DPCPX also revealed two additional peaks, namely the translocation peak at − 72 pA and an intercalation peak at − 51 pA (Fig. 4a *vs* c). The intercalation peak at − 51 pA had a low population of events (21%) whereas the translocation peak was broader and had a similar proportion to the translocation peak of CPA. The ΔNAC translocation peak disappeared with the addition of DPCPX; instead, two peaks with similar proportion of events were observed at − 24 and − 39 pA, representing the bumping and intercalation peaks, respectively (Fig. 4d *vs* f). Lastly, the C-terminus histogram of DPCPX indicated a decrease of the bumping events from 20% to 7%, an emergence of an intercalation peak at − 31 pA, and a significant decrease of the translocation peak from 77% to 38% (Fig. 4g *vs* i). For convenience, all the blockade intensities and populations events are shown in Table 2.

**Table 2:** Summary of the intensity and the population of current blockades for all the domains in the absence or presence of CPA or DPCPX

Domain-drug complex	*I*_Trans_	P_Trans_	*I*_Inter_	P_Inter_	*I*_Bump_	P_Bump_
N-term	–	–	–	–	− 30 ± 1 pA	70% ± 2%
N-term + CPA	−66 ± 6 pA	35% ± 3%	–	–	− 26 ± 1 pA	46% ± 2% [***]
N-term + DPCPX	−72 ± 7 pA	36% ± 10%	− 51 ± 2 pA	21% ± 12%	− 30 ± 2 pA	41% ± 1% [***]
C-term	−69 ± 2 pA	77% ± 4%	–	–	− 30 ± 2 pA	20% ± 3%
C-term + CPA	−66 ± 3 pA	70% ± 8% [ns]	–	–	− 38 ± 4 pA	23% ± 3% [ns]
C-term + DPCPX	−64 ± 1 pA	38% ± 2% [***]	− 31 ± 1 pA	33% ± 4%	− 19 ± 1 pA	7% ± 1% [ns]
ΔNAC	−86 ± 2 pA	58% ± 2%	–	–	− 27 ± 1 pA	19% ± 4%
ΔNAC + CPA	–	–	− 36 ± 3 pA	66% ± 3%	–	–
ΔNAC + DPCPX	–	–	− 39 ± 1 pA	35% ± 5%	− 24 ± 2 pA	34% ± 3%

Mean ± SEM. *I* and P represent intensity and population of the current blockade, respectively

ns, non-significant

*P* > 0.05, *P < 0.05, **P < 0.01, and ***P < 0.001 *vs* N-terminus or C-terminus alone (one-way ANOVA, followed by Student–Newman–Keuls multiple comparison test)

**Fig. 5:**
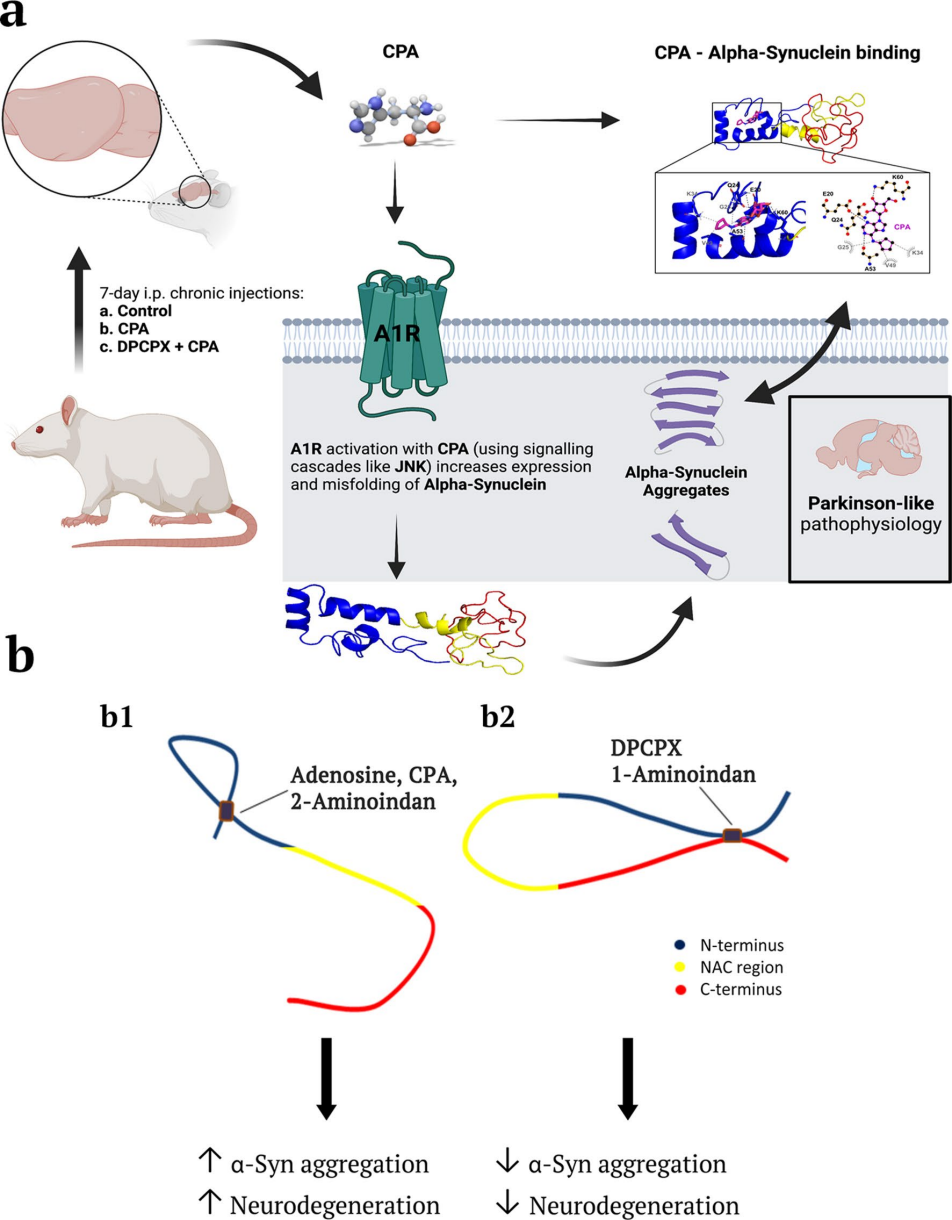
Effects of adenosine A1 receptor (A1R) ligands on α-synuclein (α-syn) expression and folding patterns in in vivo and in vitro studies. **a** A1R agonist CPA (and adenosine) increases α-syn expression and aggregation in the rat substantia nigra. Nanopore analysis and molecular docking simulations predicted binding of A1R agonist CPA (and adenosine) to the N-terminus of α-syn, leaving the NAC domain intact and able to promote aggregation. **b** (**b1**) Adenosine, CPA and 2-aminoindan bind to and stabilize α-syn to adopt a “knot” conformation which has been shown to induce aggregation and neurodegeneration. In contrast (**b2**), DPCPX and 1-aminoindan bind to both the N- and C-termini of α-syn, which does not promote aggregation and neurodegeneration. Created using BioRender.com

The changes in the shape of histograms (Figs. 2 and 4) upon addition of a drug demonstrated drug binding. Further, the increase in bumping events and decrease in translocation events demonstrate that the drug caused protein folding. Nanopore analysis showed that the drug binding resulted in either a “knot” or a “loop” α-syn conformation (Fig. 5). Interestingly, we observed intermediate gaussian peaks suggestive of the presence of a particular partially folded structure though we could not infer what the structure was (Fig. 2d–f). In other cases, there were many intermediate events that suggest the presence of many different structures (Fig. 4a, d–f). However, there may be other drug/α-syn interactions that are transient or dependent on initial drug concentrations, which could alter the proportions of translocation, bumping and intermediate events as previously reported for the drug Rasagiline [36]. Since α-syn is an intrinsically disordered protein, it is assumed to have an infinite number of conformations. Therefore, it is possible that some of these α-syn structures (bound or unbound by ligands) could minimally be present in our nanopore recordings, and consequently had non-negligible contributions to the current histograms that were not covered by the gaussian fitting in Fig. 2a, b, d–f and Fig. 4a, b, d–f. Taken together, the altered translocation and bumping (both blockade current peaks and populations of events) and the appearance of intercalation events in the N-terminus, ΔNAC, and C-terminus domains of α-syn indicate that DPCPX binds to both the N- and C-termini of α-syn.

### Molecular docking simulations reveal interactions of DPCPX and 1-aminoindan with N- and C-termini of α-syn

To confirm the biophysical results from nanopore analysis, we further characterized the α-syn–drug complexes by performing molecular docking studies of the three A1R ligands as well as 1- and 2-aminoindan with α-syn. Conformational ensemble of α-syn in solution as determined by discrete molecular dynamics simulations and further confirmed by far-UV circular dichroism and cross-linking mass spectrometry, has recently revealed stable monomeric α-syn clusters of structure [47] (C1-C8, see Additional file 1: Appendix 1). We used these structures in the molecular docking simulations to determine if any of these structures correlates with the drug/α-syn complex as predicted from the nanopore analysis. The 8 clusters of structural subpopulations have the following features: C1 structure forms a dimer and plays a role in fibril formation; C2 and C3 are precursors for oligomer formation; C3 has antiparallel β-sheets in the aggregate-prone NAC segment and forms oligomers that could be toxic to neurons; C4 has low propensity for α-helical structure like cluster C3 and, hence, is unlikely to be membrane-bound; some C5 structures interact with membranes and might be important for synaptic functions, while other C5 structures form tetramers in vivo, which are believed to promote protection against neurodegenerative disorders; C6 N-terminal residues adopt an α-helical structure that targets and anchors α-syn to membrane of synaptic vesicles; C7 is similar to C1, C3 and C4, having low propensity for α-helical formation (i.e., higher β-strand propensity), hence, α-syn monomers are likely in aqueous solution; and C8 structure plays a role in fibril formation and has high α-helix propensity like C2, C5 and C6 [47].

**Fig. 6:**
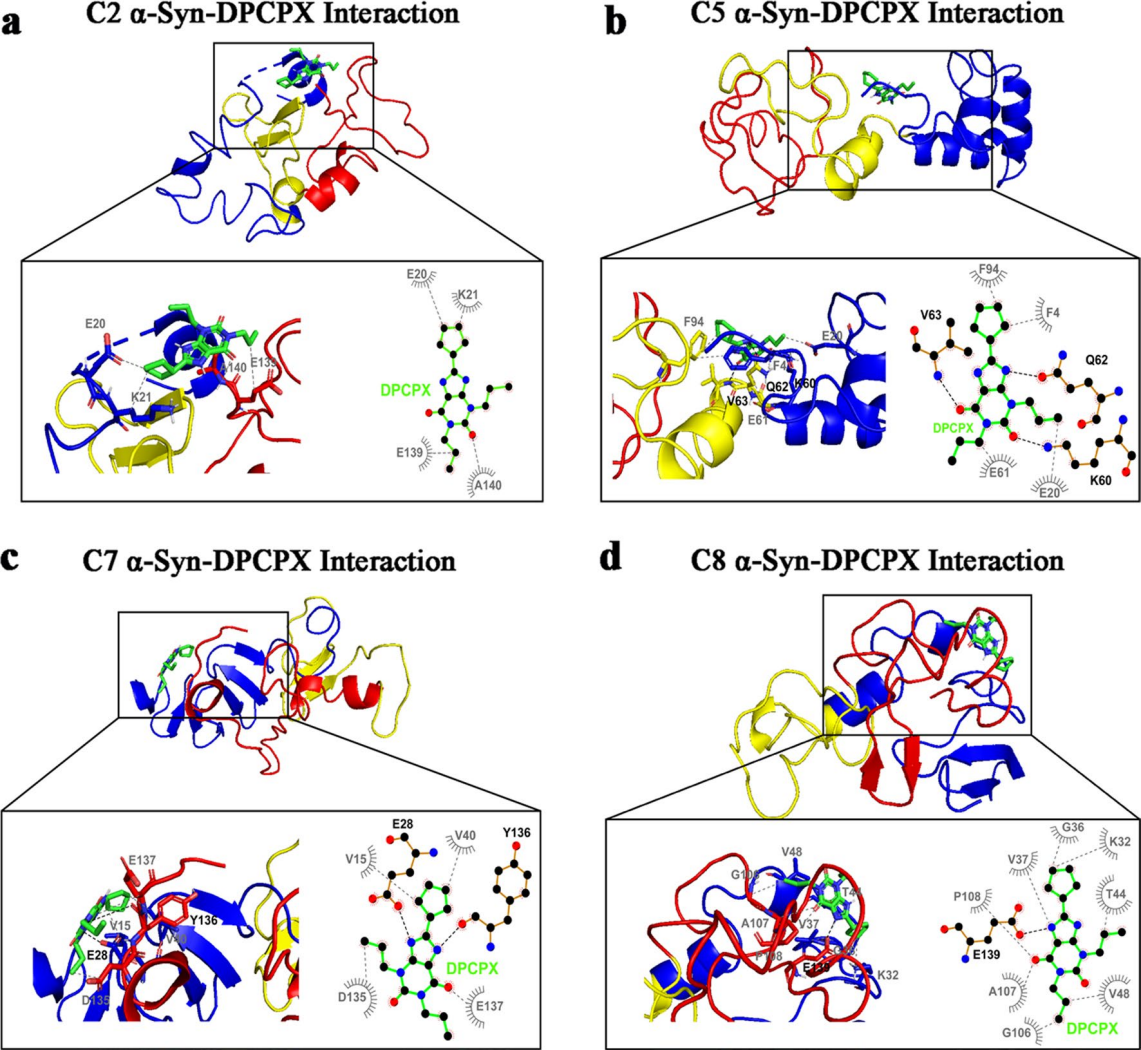
Molecular docking simulation of α-synuclein (α-syn) structures C2 (**a**), C5 (**b**), C7 (**c**), and C8 (**d**) bound to DPCPX. Below full 3D representations show magnified binding pocket of α-syn and the locations of amino acid residues responsible for each drug binding. Bold black dashed lines and amino acid residues indicate hydrogen bonding, while the grey dashed lines and amino acid residues indicate hydrophobic interactions. Hydrogen bonding of DPCPX with both the N- and C-terminal amino acid residues is observed in C7 α-syn structure (**c**). DPCPX also forms hydrogen bond with either the N-terminal (C5 α-syn structure in **b**) or C-terminal amino acids (C8 α-syn structure in **d**) and also hydrophobic bonds with portions of the NAC region. N-and C-terminal binding of DPCPX is also observed without hydrogen bonding (C2 α-syn structure in **a**). The molecular docking study was carried out using Autodock Vina module implemented in PyRx tool. Protein and ligand interactions were analyzed and visualized through Pymol and LigPlot +

Four of the eight structures of α-syn were selected to study the α-syn–DPCPX drug complex: C2, C5, C7 and C8 [47]. For the C2 structure of α-syn, DPCPX showed hydrophobic interaction with the N-terminus of α-syn, specifically the negatively charged glutamic acid 20 (E20) and positively charged lysine 21 (K21), and with the very end of the C-terminus (amino acids E139 and alanine 140 (A140)) (Fig. 6a). However, for the C5 structure (Fig. 6b), DPCPX was shown to form hydrophobic bonds with the N-terminus (amino acids phenylalanine 4 (F4) and E20) and hydrogen bonds with K60, and hydrophobic bonds with the NAC region (amino acids E61 and F94). DPCPX also formed hydrogen bonds with the NAC region (amino acids glutamine 62 (Q62) and valine 63 (V63)). Similar as the interactions with C2, DPCPX binding to the N-terminus (hydrogen bond with E28, with additional hydrophobic interactions with V15 and V40) and the C-terminus (hydrogen bond with tyrosine 136 (Y136) with additional hydrophobic interactions with aspartic acid 135 (D135) and E137) of the C7 structure was revealed (Fig. 6c). Furthermore, DPCPX was shown to bind to the C8 structure in the N-terminus (amino acids K32, glycine 36 (G36), V37, threonine 44 (T44) and V48) and the C-terminus with a hydrogen bond at the E139 and additional hydrophobic interactions at G106, A107 and proline 108 (P108). For all the structures C2, C5, C7 and C8, DPCPX was revealed to reside within a closed globular conformation and interacts with both the N- and C-termini of α-syn. However, it is also possible that other α-syn structures can bind DPCPX via hydrogen bonding with the N-terminus (H50 and K12 of C3 structure) or C-terminus (E137 of C1 structure) and hydrophobic interactions with the NAC region (Additional file 1: Fig. S2).

**Fig. 7:**
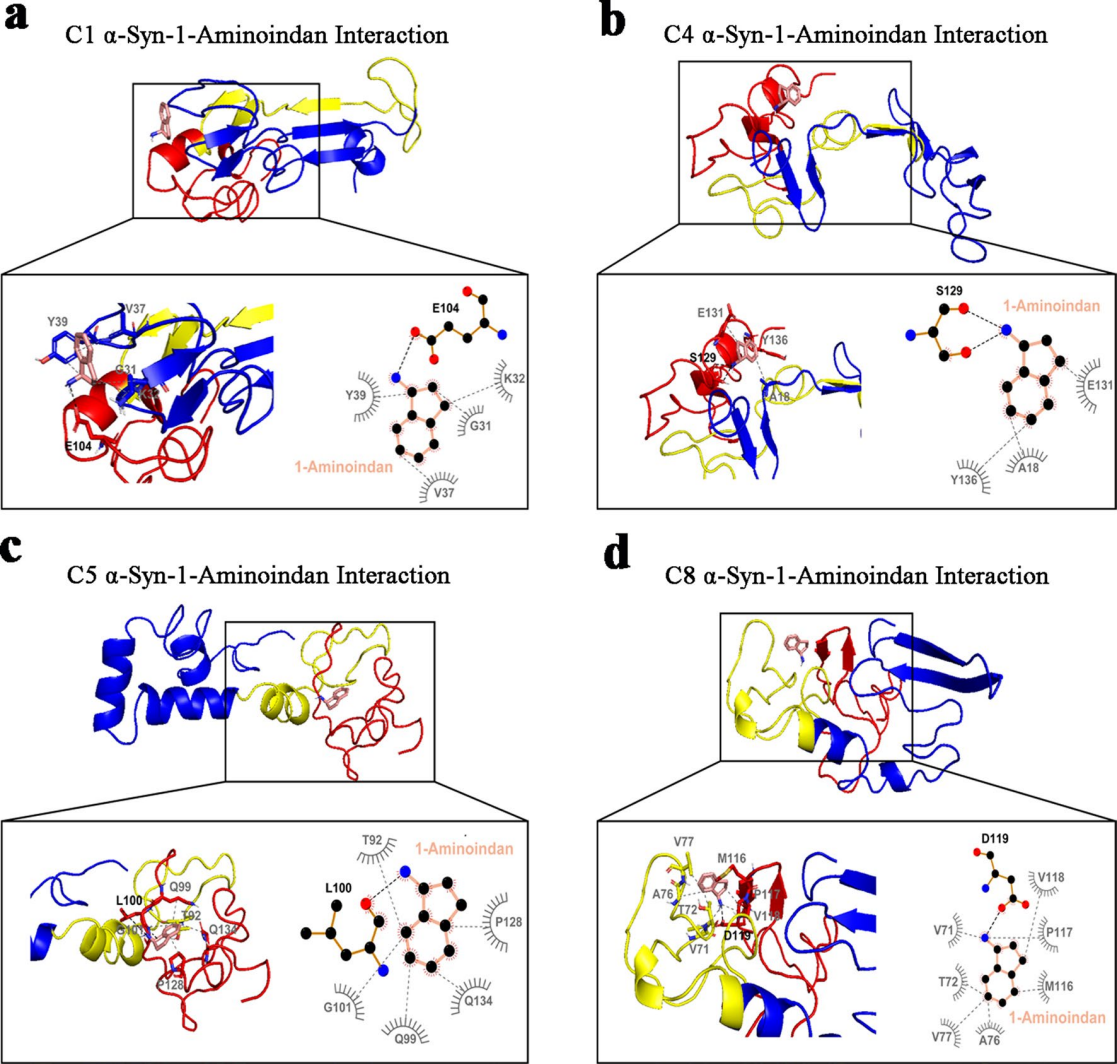
Molecular docking simulation of α-synuclein (α-syn) structures C1 (**a**), C4 (**b**), C5 (**c**) and C8 (**d**) bound to 1-aminoindan. Below full 3D representations show the magnified binding domains of α-syn and the amino acid residues in both the N- and C- termini of α-syn that facilitate drug binding. Bold black dashed lines and amino acid residues indicate hydrogen bonding, whereas the grey dashed lines indicate hydrophobic interactions. Hydrogen bonding of 1-aminoindan with C-terminal amino acid residues is observed in C1, C4, C5 and C8 α-syn structures. In addition, hydrophobic interactions occur between 1-aminoindan and N-terminal amino acid residues (C1 and C4 α-syn structures) and also between 1-aminoindan and portions of the NAC region (C5 and C8 α-syn structures). The molecular docking study was carried out using Autodock Vina module implemented in PyRx tool. Protein and ligand interactions were analyzed and visualized through Pymol and LigPlot +

Like DPCPX, four α-syn structures, C1, C4, C5 and C8, were selected to analyze the binding mode of 1-aminoindan to α-syn. For the C1 structure, 1-aminoindan was shown to form hydrogen bond with E104 of the C-terminus as well as hydrophobic interactions with the aromatic positively charged cleft of the N-terminus (amino acids G31, K32, V37, and Y39) (Fig. 7a). For the C4 structure (Fig. 7b), 1-aminoindan was shown to form hydrogen bonds with the polar serine 129 (S129) and hydrophobic interactions with E131 and Y136 of the C-terminus, and interact with the N-terminus of C4 structure, forming hydrophobic bonds with A18. These indicate the formation of a loop conformation between the N- and C-termini of the protein when 1-aminoindan binds to the C4 structure. However, the C5 and C8 structures were shown to form different drug-protein complexes that may regulate tetramer formation (C5) or fibrillation (C8). 1-Aminoindan appeared to bind only to the C-terminal polar cleft between T92, Q99 and Q134 of the C5 structure (Fig. 7c). Also, 1-aminoindan was shown to form further hydrophobic bonds with G101 and P128 and a hydrogen bond with L100. Lastly, 1-aminoindan had hydrophobic interactions with the NAC region of the C8 structure (amino acids V71, T72, A76, and V77), and in the C-terminus (amino acids methionine 116 (M116), P117 and V118) (Fig. 7d). 1-Aminoindan also formed a hydrogen bond with D119 in the C-terminus of the structure.

These results indicated that the A1R antagonist DPCPX and Rasagiline metabolite 1-aminoindan possess similar binding interactions with α-syn. As the full crystal structure of α-syn is not yet available, we suggest that using the four conformations of α-syn in our molecular docking studies could provide more complete information on the binding interactions of DPCPX and 1-aminoindan with α-syn.

### Molecular docking simulations reveal that CPA, adenosine and 2-aminoindan bind to the N-terminus of α-syn

**Fig. 8:**
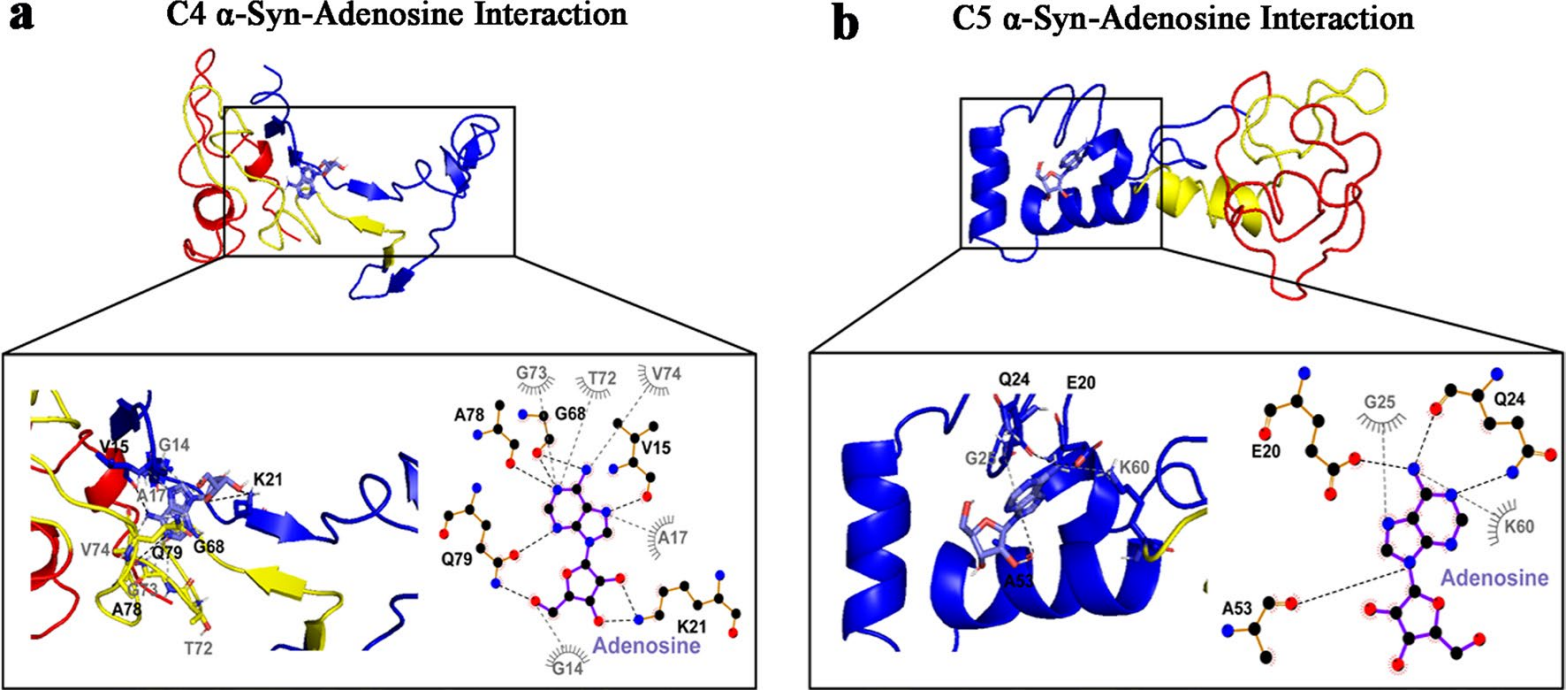
Molecular docking simulation of α-synuclein (α-syn) structures C4 (**a**) and C5 (**b**) bound to adenosine. Below full 3D representations show the magnified binding pocket of α-syn and the amino acid residue locations responsible for each drug binding. Bold black dashed lines and amino acid residues indicate hydrogen bonding, whereas the grey dashed lines and amino acid residues indicate hydrophobic interactions. Adenosine only formed hydrogen bonds and hydrophobic interactions with N-terminal amino acid residues in C5 α-syn structure. In addition, adenosine also formed hydrogen bonds with amino acid residues within the N-terminus and NAC region in C4 α-syn structure. The molecular docking study was carried out using Autodock Vina module implemented in PyRx tool. Protein and ligand interactions were analyzed and visualized through Pymol and LigPlot +

Based on the results of nanopore analysis, molecular docking simulation of α-syn binding to adenosine, A1R agonist CPA, and methamphetamine analog 2-aminoindan was conducted. Results showed that adenosine mainly bound to the N-terminus of α-syn (blue alpha-helix region) (Fig. 8). For the C4 structure, adenosine formed hydrogen bonds with V15 and K21 in the positively charged cleft in the C4 N-terminus and with G68, A78 and Q79 in the C4 NAC region. Other hydrophobic interactions were revealed at G14 and A17 in the N-terminus and at T72, G73 and V74 in the NAC region. For the C5 structure, adenosine bound only to the N-terminus, forming hydrogen bonds with the polar negatively charged cleft of the N-terminus (amino acids E20, Q24, and A53) and hydrophobic interactions with G25 and K60.

**Fig. 9:**
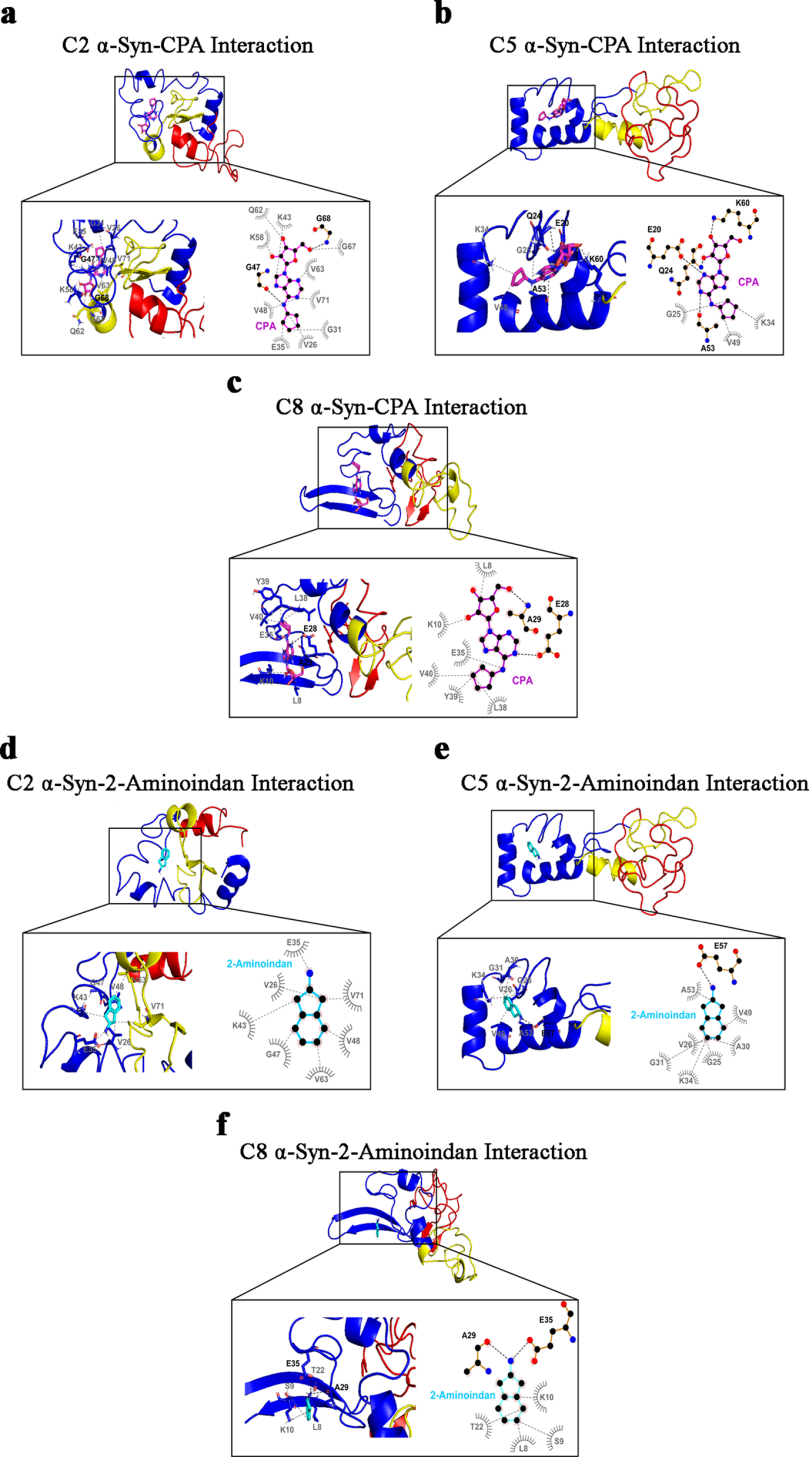
Molecular docking simulation of α-synuclein (α-syn) structures C2 (**a**), C5 (**b**), and C8 (**c**) bound to CPA; C2 (**d**), C5 (**e**), and C8 (**f**) bound to 2-aminoindan. Below full 3D representations show the magnified binding pocket of α-syn and the amino acid residue locations responsible for each drug binding. Bold black dashed lines and amino acid residues indicate hydrogen bonding, whereas the grey dashed lines and amino acid residues indicate hydrophobic interactions. Both CPA and 2-aminoindan formed hydrogen bonds and hydrophobic interactions with the N-terminal amino acids only (C5 and C8 α-syn structures) (**b**-**c** and **e**–**f**, respectively). CPA also forms hydrogen bond and hydrophobic interactions with amino acids within the N-terminal and the NAC region (C2 α-syn structure) (**a**). In contrast, 2-aminoindan only forms hydrophobic interactions with the N-terminus and NAC domain in C2 α-syn structure (**d**). The molecular docking study was carried out using Autodock Vina module implemented in PyRx tool. Protein and ligand interactions were analyzed and visualized through Pymol and LigPlot +

CPA is a chemical derivative of adenosine that shows greater selectivity as an A1R agonist, and thus is expected to interact with α-syn similarly as adenosine. Similar to adenosine, CPA formed hydrogen bonds with G47 of the N-terminus and G68 of the NAC region of the C2 α-syn structure (Fig. 9a). CPA also had various hydrophobic interactions in the N-terminus (amino acids V26, G31, E35, K43, V48, and K58) and in the NAC region (amino acids Q62, V63, G67, and V71). For the C5 structure, CPA formed a hydrogen bond with the negatively charged E20, as well as the positively charged K60, Q24 and A53 in the N-terminus (Fig. 9b). It also formed hydrophobic interactions with G25, K34 and V49. For the C8 structure, CPA interacted with similar negatively charged N-terminal cleft containing E28 and A29 (Fig. 9c), and also formed various hydrophobic bonds in the N-terminus (amino acids L8, K10, E35, L38, Y39, and V40).

Similar to CPA, 2-aminoindan was also shown to have various hydrophobic interactions with the N-terminus (amino acids V26, E35, K43, G47, and V48) and the NAC region (amino acids V63, and V71) of the C2 structure of α-syn (Fig. 9d). Moreover, 2-aminoindan also formed a hydrogen bond with E57 and hydrophobic interactions with other residues within the N-terminus of the C5 structure (amino acids G25, V26, A30, G31, K34, V49, and A53) (Fig. 9e). Similar to CPA, 2-aminoindan formed hydrogen bonds with A29 and E35 and hydrophobic interactions with other residues inside the N-terminus of the C8 structure (amino acids L8, Serine 9 (S9), K10, and T22) (Fig. 9f).

Taken together, the molecular docking studies confirmed the results of nanopore analyses, that adenosine, CPA and 2-aminoindan mainly interacted with the N-terminus of α-syn (C5 cluster); however, other subpopulations of α-syn clusters showed hydrogen bonding of CPA and 2-aminoindan with other N-terminal residues only (C8) or hydrogen bonding of adenosine with the N-terminal and proximal NAC amino acid residues (C4). The interactions of the drugs with α-syn N-terminus are expected to promote α-syn aggregation. In contrast, DPCPX and 1-aminoindan showed binding to both N- and C-terminal regions of α-syn, which may promote an α-syn conformation that prevents α-syn aggregation.

### Comparison of α-syn binding of adenosine with that of other standard nucleosides using molecular docking simulation

Adenosine is not only an endogenous agonist of all the purinergic G protein-coupled receptors, but also a purine ribonucleoside [55]. To determine whether adenosine binds specifically to α-syn, we performed additional molecular docking simulations to compare α-syn binding of adenosine with that of the four other standard nucleosides (guanosine, cytidine, thymidine, and uridine) and the adenosine metabolite inosine, using the C5 α-syn structure (Additional file 1: Fig. S3).

All the purine nucleosides were shown to bind to a similar hydrophobic pocket in the N-terminus of the C5 α-syn conformation. Similar to adenosine, almost all of them formed hydrogen bonds with the positively charged K34 (inosine, guanosine, thymidine, and uridine) and with the negatively charged E20 (guanosine, cytidine, and uridine) (Additional file 1: Fig. S3). Inosine had similar hydrophobic interactions with Q24, G25 and A53 as adenosine, and formed additional hydrophobic bonds with C5 α-syn conformation at H50 and E57. Inosine formed hydrogen bonds with V49 and T54 in the N-terminus as well. Guanosine formed hydrogen bonds with the polar cleft of Q24 and A53 of the N-terminus same as adenosine (Additional file 1: Fig. S3, Fig. 8b). Conversely, cytidine (Additional file 1: Fig. S3c) interacted with the same amino acids, but through hydrophobic interactions (amino acids A19, Q24, G25, A53 and E57). Moreover, thymidine and uridine shared the same hydrophobic interactions with the C5 α-syn structure (Additional file 1: Fig. S3d, c), and both bound to G25, A53 and E57. Uridine also formed other hydrophobic bonds with A19 and Q24, like cytidine (Additional file 1: Fig. S3c, e).

### A1R agonist CPA and drugs that bind to α-syn N-terminus increase α-syn expression and aggregation in SN and hippocampal neurons

**Fig. 10:**
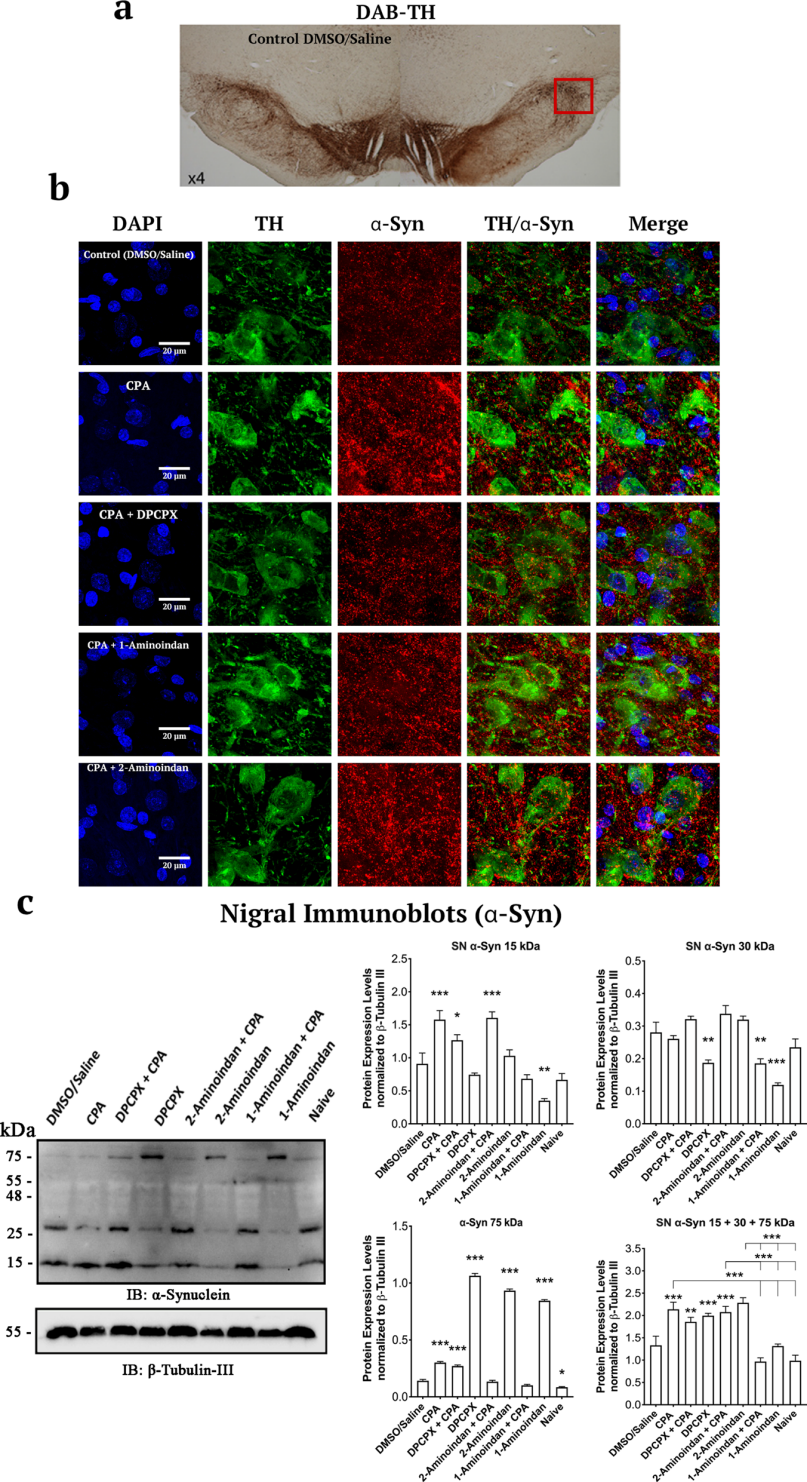
Summary of the surface area analysis of the pars compacta region of the substantia nigra for DAPI, tyrosine hydroxylase (TH), and α-synuclein (α-syn). (**a**) Image of a 40-μm nigral brain slice in the DMSO/Saline control group, with 3,3’-diaminobenzidine (DAB) and TH staining at 4 × magnification with a light microscope. (**b**) Representative images of DAPI (Blue), TH (Green, Alexa Fluor 555), and α-syn (Red, Alexa Fluor 647) staining in the substantia nigra pars compacta of rats with 7-day chronic intraperitoneal injections of the following agents: Control (DMSO/Saline), CPA, DPCPX + CPA, 1-aminoindan + CPA, and 2-aminoindan + CPA. CPA with or without 2-aminoindan increased α-syn immunofluorescence compared to control. The CPA-induced increase in α-syn was attenuated by DPCPX or 1-aminoindan. Scale bar, 20 μm. (**c**) Western blots from total lysates of the substantia nigra and quantification of α-syn level in the substantia nigra. CPA increased the level of 15 kDa α-syn monomers, which was attenuated by DPCPX and 1-aminoindan but not by 2-aminoindan. DPCPX and 1-aminoindan alone significantly reduced the level of 30 kDa α-syn dimers. In contrast, DPCPX, 2-aminoindan, and 1-aminoindan alone significantly increased the 75 kDa α-syn, which likely represent the α-syn tetramers. All values were normalized to β-tubulin III. *n* = 4 animals in each treatment group. Mean ± SEM. ns, non-significant; *P < 0.05; **P < 0.01; and ***P < 0.001 (one-way ANOVA followed by Student–Newman–Keuls post-hoc multiple comparison test)

**Fig. 11:**
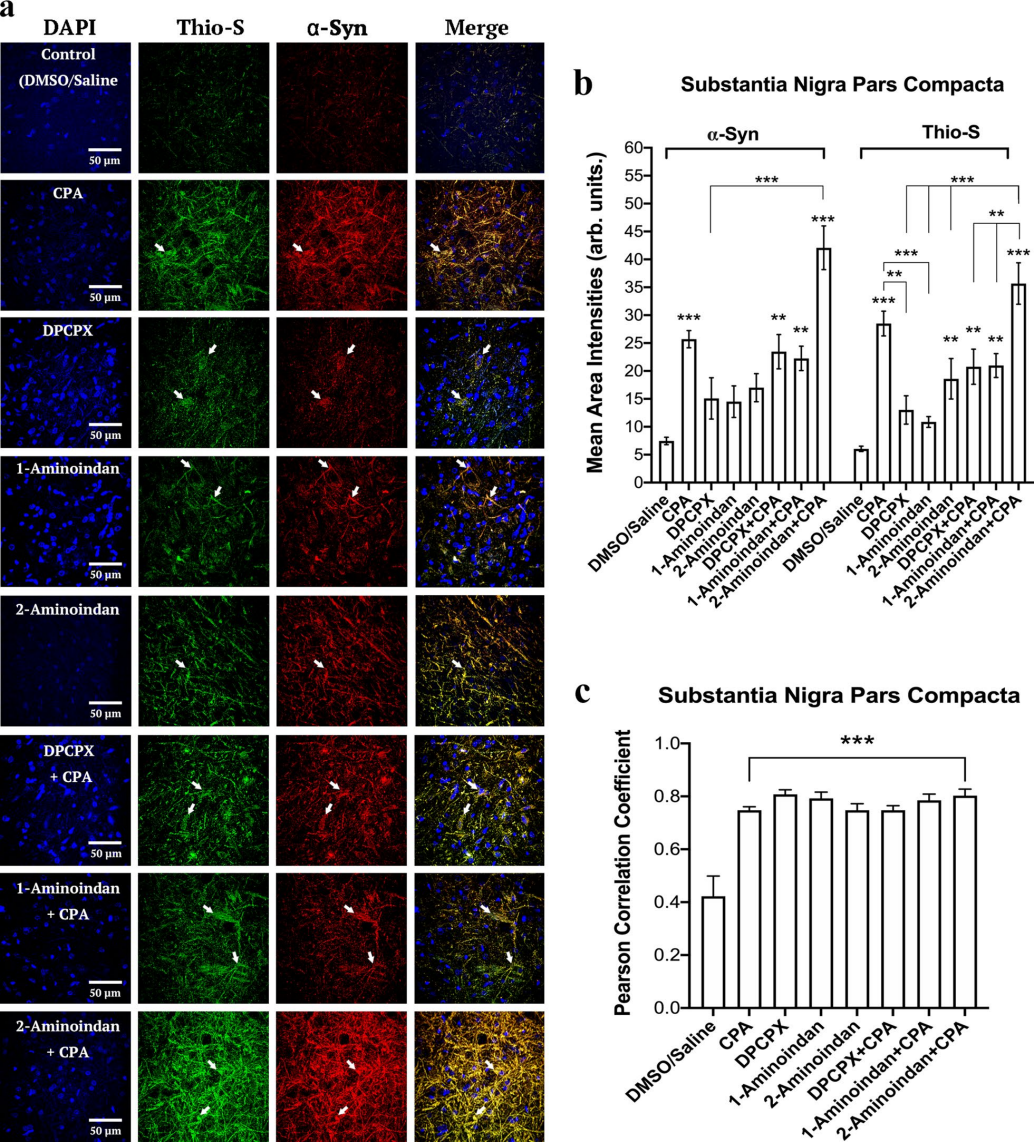
Summary of surface area analysis of α-synuclein (α-syn) and Thioflavin S in the substantia nigra (SN) pars compacta region. (**a**) Confocal microscopic images of DAPI, α-syn and Thioflavin S staining in 40-μm nigral brain slices of rats with the following treatments: Control (DMSO/Saline), CPA, DPCPX, 1-aminoindan, 2-aminoindan, DPCPX + CPA, 1-aminoindan + CPA, and 2-aminoindan + CPA. Scale bar, 50 μm. (**b**) The mean area intensities of α-syn and Thioflavin S in the SN pars compacta. The fluorescence intensity was quantified in a 100 × 100 μm^2^ region and normalized by subtracting the fluorescence intensity in a 50 × 50 μm^2^ background non-cell body bottom area. CPA increased the levels of α-syn and aggregated α-syn, and these levels were further enhanced by co-treatments with 2-aminoindan. (**c**) Pearson correlation coefficient of α-syn and Thioflavin S in the SN pars compacta with CPA. Average intensity values and correlation coefficients in bars represent mean ± SEM from *n* = 4 independent experiments. ns, non-significant; *P < 0.05; **P < 0.01; and ***P < 0.001 (one-way ANOVA followed by Student–Newman–Keuls post-hoc multiple comparison test)

To investigate whether drug binding to the N- and/or C-terminus of α-syn can affect the levels of α-syn expression and aggregation in vivo, we administered the drugs individually or in combination with the A1R agonist CPA. Representative images of the SN pars compacta region labelled with DAPI, TH, and α-syn showed that α-syn was localized in the soma (cytosol, nuclei) and presumably the dendrites of dopaminergic neurons (Fig. 10b). Interestingly, α-syn expression was increased by CPA in the absence or presence of DPCPX, 1-aminoindan, or 2-aminoindan (Fig. 10b, and 11a, b) compared to the control (0.1% DMSO in 0.9% saline). The 2-aminoindan + CPA treatment induced the highest level of α-syn protein in the SN pars compacta (Fig. 11a, b). In contrast, DPCPX, 1-aminoindan or 2-aminoindan alone did not significantly increase the α-syn protein level (Fig. 11a, b).

To determine whether these changes in α-syn protein level correlated with the level of α-syn aggregation, the SN pars compacta was co-labelled with α-syn marker and Thio-S. As shown in Fig. 11a-c, treatments with CPA, DPCPX + CPA, 1-aminoindan + CPA, and 2-aminoindan + CPA all increased the Thio-S level. Treatment with 2-aminoindan alone also enhanced Thio-S labelling, and co-administration of 2-aminoindan with CPA caused a significant further elevation of Thio-S compared to 2-aminoindan alone (Fig. 11b). In contrast, treatments with DPCPX or 1-aminoindan alone did not significantly increase Thio-S labelling or attenuate the CPA-induced increase in Thio-S level. The colocalization of Thio-S signal with α-syn was increased in all the treatments compared to control (about a two-fold increase in Pearson correlation coefficients, Fig. 11c). In conclusion, our results showed that 7-day systemic administration of CPA, alone or in combination with 2-aminoindan, increased expression and aggregation of α-syn in the SN pars compacta (Figs. 10 and 11).

A1R is widely distributed in other regions of the brain including the hippocampus. Therefore, the CA1 region of the hippocampus was also analyzed for α-syn expression and aggregation. Similar to the nigral tissue, treatments with CPA and 2-aminoindan + CPA increased the levels of both α-syn and Thio-S (Additional file 1: Fig. S4a). In particular, CPA and 2-aminoindan + CPA induced a four-fold increase of α-syn compared to the control (Additional file 1: Fig. S4b). This increase was also observed for colocalization of Thio-S with α-syn (Additional file 1: Fig. S4c). However, co-administration of CPA with DPCPX or 1-aminoindan caused significant attenuation of α-syn accumulation compared to CPA treatment (Additional file 1: Fig. S4b, c).

### Western blotting analysis of α-syn in SN

Treatment with the A1R agonist CPA and 2-aminoindan + CPA induced a significant 1.5-fold increase in the level of monomeric α-syn (15 kDa band) in SN; however, the 1-aminoindan treatment group showed an approximately three-fold decrease in α-syn compared to the DMSO/saline control group (Fig. 10c). The CPA-induced increase in α-syn was partially attenuated by co-administration with DPCPX and was fully restored to control levels by 1-aminoindan co-treatment. Prominent signals for 30 kDa α-syn were shown in the SN immunoblots, but not detectable in the hippocampal lysate immunoblots (data not shown). In contrast to the monomeric 15 kDa α-syn band, the 30 kDa α-syn signal was not significantly altered by CPA, DPCPX + CPA, 2-aminoindan + CPA, or 2-aminoindan treatment, compared to control (DMSO/saline) or naive group; however, the 30 kDa α-syn signal was significantly decreased by DPCPX (*P* < 0.01), 1-aminoindan + CPA (*P* < 0.01), and 1-aminoindan alone (*P* < 0.001) treatments. Interestingly, the level of 75 kDa α-syn was significantly higher in the DPCPX, 2-aminoindan or 1-aminoindan treatment alone group, compared to CPA, CPA + DPCPX, or control treatment (*P* < 0.001).

Moreover, when the 15 kDa, 30 kDa and 75 kDa α-syn densitometry values were added, we still detected significantly higher levels of total α-syn in the CPA (*P* < 0.01), DPCPX + CPA (*P* < 0.033), and 2-aminoindan + CPA (*P* < 0.001) groups (Fig. 10c). It is noteworthy that significant accumulation of α-syn in the SN lysate was associated with treatments with compounds that were found to bind only to the N-terminus of α-syn (i.e., the A1R agonist CPA and 2-aminoindan); moreover, this elevation could be attenuated by co-treatments with compounds that were found to bind to both the N- and C-termini of α-syn (e.g., DPCPX, 1-aminoindan). In addition, the observed higher molecular weight band at 75 kDa likely indicates the presence of C5 α-syn structures that differentially bind to DPCPX (Fig. 6b), 1-aminoindan (Fig. 7c), and 2-aminoindan (Fig. 9e), since the C5 structure of α-syn is known to be a precursor for the formation of tetramers [47].

### CPA and 2-aminoindan increase neurodegeneration of SN pars compacta dopaminergic neurons and hippocampal pyramidal neurons

**Fig. 12:**
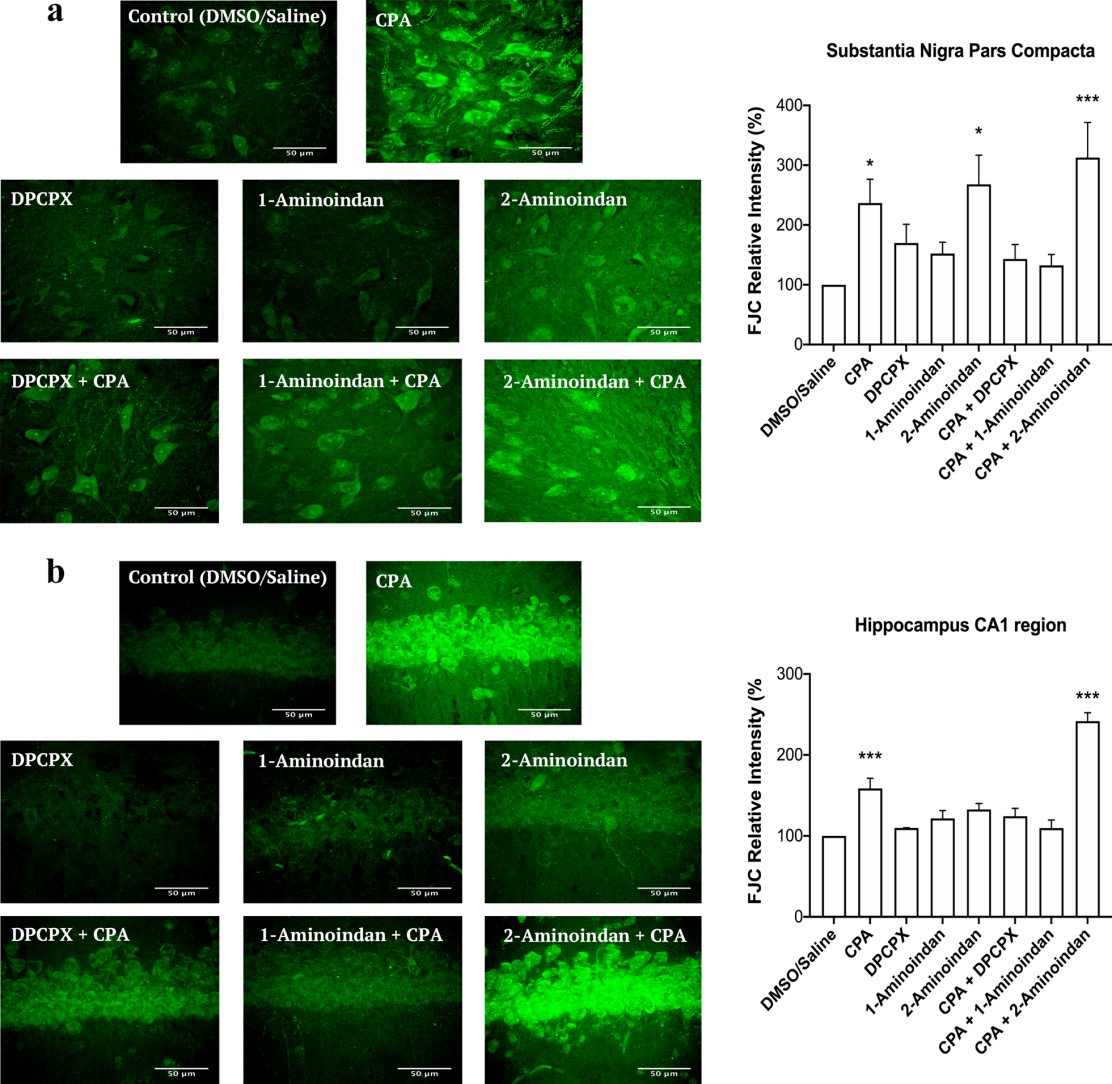
Fluoro-Jade C (FJC) staining in the SN pars compacta (**a**) and CA1 of hippocampus (**b**) of rats with 7-day chronic intraperitoneal injection of Control (DMSO/saline), CPA, DPCPX, 1-aminoindan, 2-aminoindan, DPCPX + CPA, 1-aminoindan + CPA, and 2-aminoindan + CPA. Scale bar 50 μm. Summary bar graphs show significant increases in the relative fluorescence intensity of FJC staining in pars compacta after CPA, 2-aminoindan, and CPA + 2-aminonindan treatments (**a**). In contrast, only CPA and CPA + 2-aminoindan treatments significantly increased FJC fluorescence in the CA1 hippocampal neurons (**b**). FJC fluorescence intensity in a 100 × 100 μm^2^ region was normalized to the control group (100%). Values are shown as mean ± SEM. The average FJC fluorescence values were obtained from *n* = 4 independent experiments. ns, non-significant; *P < 0.05; **P < 0.01; and ***P < 0.001 (one-way ANOVA followed by Student–Newman–Keuls post-hoc multiple comparison test)

Having shown that CPA and 2-aminoindan alone or in combination can increase α-syn aggregation, we then determined whether these treatments could lead to neuronal damage. We used FJC as a common fluorescent marker for neurodegeneration in the CNS [31]. FJC staining was performed in nigral slices −5.30 to −5.60 mm from the bregma as well as hippocampal slices −3.80 to −4.16 mm from the bregma. Representative high-magnification images of FJC staining in the pars compacta region of SN indicate that CPA alone, 2-aminoindan alone, and 2-aminoindan + CPA co-administration all increased the level of FJC fluorescence (Fig. 12a). In contrast, DPCPX or 1-aminoindan alone did not significantly increase FJC staining, but both were effective in attenuating the CPA-induced increase in neurodegeneration (Fig. 12a). Similar results were observed in the CA1 region of the hippocampus, except that 2-aminoindan alone did not cause significant neurodegeneration (Fig. 12b). CPA and 2-aminoindan + CPA treatments demonstrated much higher levels of degenerating pyramidal neurons compared to the other treatments. Similar to the nigral slices, the co-administration of DPCPX or 1-aminoindan with CPA prevented neurodegeneration in hippocampal slices.

Taken together with the above results from nanopore analysis, molecular docking and Thio-S labelling, these results suggest that compounds that bind to both N- and C-termini of α-syn (e.g., DPCPX and 1-aminoindan) may be effective in attenuating the neurotoxic effects of compounds that bind to α-syn and promote α-syn accumulation and misfolding (e.g., CPA, adenosine and 2-aminoindan).

## Discussion

One of the most commonly used treatments for PD patients is Levodopa (a dopamine precursor). However, this treatment is associated with numerous adverse effects such as early loss of voluntary movement, severe dyskinesia episodes and most predominantly end-of-dose worsening [56, 57]. Previous studies on dopamine receptor agonists, catechol-O-methyltransferase antagonists, monoamine oxidase inhibitors, and antagonists of dopamine transporters have shown promising results in slowing the progression and alleviating the symptoms of the disease [58–61]. However, these therapies are unable to prevent PD progression without causing other significant side effects including an increased risk of cardiac-valve regurgitation, hypertension, confusion and hallucinations [62].

Adenosine binds to its inhibitory A1Rs (coupled to G_αi_) and its excitatory A2A receptors (A2AR, coupled to G_αs_), and A2ARs are believed to contribute to the pathogenesis of PD, which has prompted the development of small molecular agents for potential PD therapy, including apedenoson, preladenant, regadenoson and SYN-115 [24, 63]. Recently, several studies have reported that A2AR antagonism produces far better results in slowing down the pathology and progression of PD and improving symptom management [24, 64–68]. Unfortunately, the majority of these drugs have failed in clinical trials except for istradefylline, which has been pursued as a potential PD drug in Phase III clinical trials in Japan [69, 70] but recently been approved by US Food and Drug Administration as the only non-dopaminergic add-on therapy for the treatment of so-called “off phenomenon” and motor fluctuations of Levodopa therapy in PD [71]. We have suggested that a possible cross-talk between A1Rs and A2ARs could contribute to PD and other neurodegenerative diseases due to the elevated levels of adenosine in the ageing brain, which may increase A2AR activation [24, 72–74]. More recently, we have reported that chronic stimulation of A1Rs with the A1R agonist CPA leads to increased expression of α-syn both in the SN region of Sprague–Dawley rat brain and in the dopaminergic MN9D cells [25]. Here, our in vitro findings suggest that drugs that bind to A1Rs may play a major role in synucleinopathy, independent of their canonical function as A1R agonist or antagonist. We show for the first time that these adenosine-related compounds could bind differentially to different regions of α-syn, and thus could contribute to α-syn protein misfolding and the development of α-synucleinopathy in PD. We observed that CPA interacted not only with the α-syn N-terminus but also with α-syn lacking the NAC region (ΔNAC). However, the A1R antagonist DPCPX significantly altered the population histograms from the nanopore analysis when the N-terminus, C-terminus or the ΔNAC regions were studied, indicating DPCPX binding to all these α-syn polypeptide domains. Based on the nanopore analysis, we propose that the two A1R ligands CPA and DPCPX have different binding interactions with α-syn. Therefore, as we previously suggested for 2-aminoindan [20, 21], CPA could bind to the N-terminus of α-syn similar to 2-aminoindan, thereby leaving the NAC domain free to misfold and cause a higher chance of protein aggregation. In contrast, DPCPX, similar to 1-aminoindan, metformin and caffeine, appeared to bind to both the N- and C-termini of α-syn, creating a “loop” conformation of the protein (Fig. 5b2) [20, 21]. This loop conformation has been suggested to prevent the NAC domain from misfolding and hence promote neuroprotection by stopping aggregation of misfolded α-syn protein fibrils and formation of Lewy bodies. Therefore, the two possible conformations adopted by α-syn in the presence of CPA (knot conformation) or DPCPX (loop conformation) (Fig. 5) are expected to contribute to increased or decreased α-syn aggregation, respectively. Additionally, the blockade currents by CPA and DPCPX resembled those by 2-aminoindan and 1-aminoindan, respectively. The metabolite of Rasagiline, 1-aminoindan, is an irreversible inhibitor of the monoamine oxidase type B enzyme which is administered in mono- and/or poly-therapeutic route to treat early symptoms of PD as well as cognitive impairments and fatigue [75, 76]. Conversely, 2-aminoindan, an amphetamine analogue, is shown to cause PD-like symptoms in long-term users and addicts [77]. Recent case reports indicated that amphetamine and methamphetamine users have a three-fold risk of developing PD compared with non-users. Interestingly, this risk is particularly high in women and it manifests even at 30 years of age [78]. Although 1-aminoindan and 2-aminoindan have very similar structures, they have been shown to hold very different blockade current properties [20, 21].

The blockade histograms for 1-aminoindan demonstrate that most events are related to translocation, as observed previously [20, 21]. In the presence of 10 μM 1-aminoindan, a broad translocation peak at − 73 pA and a small bumping peak at − 34 pA were observed. Nanopore analysis with the use of different α-syn domains suggests that 1-aminoindan binds to both the N- and C-termini of α-syn, and by doing so, the drug-protein complex is expected to adopt a “loop” conformation that promotes neuroprotection by preventing α-syn misfolding. Our results from both nanopore analysis and molecular docking suggest that DPCPX resembles 1-aminoindan in binding pattern and drug-protein conformation. Conversely, our previous report has also established that the 2-aminoindan histograms have two peaks with similar proportions of events, namely, the proportion of translocation events at 48% and bumping events at 40% [20, 21]. However, 2-aminoindan appears to bind with higher affinity to α-syn due to the high binding constant 5 × 10^4^ M^−1^ derived from isothermal titration calorimetry (ITC), and most importantly, because it binds only to the N-terminus of the protein. This N-terminal binding of 2-aminoindan has been suggested to promote a neurotoxic “knot” conformation that will lead to increased α-syn aggregation. Here, the nanopore analysis using full-length and different domains of α-syn combined with molecular docking attempts using aqueous, membrane-unbound α-syn structures [47] showed that both adenosine and the selective A1R agonist CPA closely resembled 2-aminoindan in the binding interactions and drug-protein conformations, indicating that these A1R agonists bind prominently to the N-terminus of α-syn. Although our results indicate a broad agreement between in vitro and in silico techniques, both methods have their own inherent limitations. Our nanopore analysis gives an excellent prediction of the compound binding site, but it does not provide precise amino acid residue(s) and positions of the drug binding. At best, our nanopore analysis indicated formation of a “knot” or “loop” conformation (Fig. 5) when the ligand-interacting residues within the N-terminus, NAC region, and/or C-terminus of α-syn are bound to promote protein folding. On the other hand, for molecular docking, the residues that bind to each ligand may be dependent on the starting model used; that is, compared to the aqueous α-syn cluster structures used in the present study, we found distinct ligand-interacting amino acid residues that were bound by CPA and DPCPX when the micelle-bound 1XQ8 model of α-syn was used (data not shown) [79]. Moreover, it appears that conformational selection may result in only one or several of the conformations used in the present study to be populated. Admittedly, the lack of site-directed mutational evidence that could help validate the docking results and directly confirm the ligand interactions, is a major weakness of the present study. Based on the molecular docking results, future studies using site-directed mutagenesis of these residues and retesting with nanopore analysis are needed to understand the conformational changes of α-syn upon binding to CPA, DPCPX, and other compounds. Taken together, our results show that CPA and DPCPX, in addition to their canonical function as *bona fide* A1R ligands, also bind to α-syn to modulate its misfolding and aggregation patterns.

Previous studies using nanopore analysis and a yeast model of PD reported that several compounds that bind to α-syn, including 1-aminoindan and the dimer compounds containing caffeine linked to 1-aminoindan, nicotine or metformin, can indeed prevent α-syn aggregation and promote survival in yeast [21, 36, 54]. Moreover, we previously reported that prolonged A1R activation in the brain produced by i.p. injections of CPA (3 mg/kg) once daily for three days led to significant neuronal loss in rat hippocampus in vivo [24]. More recently, we also reported that longer-term A1R stimulation with 5 mg/kg CPA (i.p. injections daily for 5 weeks) led to increased α-syn expression and accumulation in the SN neurons, which was associated with motor and cognitive deficits in Sprague–Dawley rats [25]. In the present study, we also showed that 7-day co-administration of either DPCPX or 1-aminoindan with CPA prevented the CPA-induced α-syn accumulation and aggregation in both SN pars compacta and hippocampal CA1 region, and this coincided with significantly reduced neurodegeneration of dopaminergic neurons in the SN pars compacta and pyramidal neurons in the hippocampus. However, future studies are required to further differentiate the precise roles of A1R ligand stimulation and α-syn–drug binding to promote or attenuate α-syn aggregation and subsequent neurodegeneration using rational mutagenesis design or using knockdown studies involving A1R or α-syn genes.

According to our previous report [25], the neurotoxic effect of CPA observed in the present study was likely mediated in part by A1R-induced downstream activation of JNK/c-Jun and sortilin-dependent binding and accumulation of α-syn in dopaminergic neurons. Since we found in the present study that CPA and DPCPX could also bind directly to α-syn, in the future, it would be important to test in A1R knockout mice or in MN9D dopaminergic neurons with CRISPR/Cas9 gene knockdown of A1Rs to determine whether chronic CPA administration could still induce upregulation and accumulation of misfolded α-syn, and also whether DPCPX or 1-aminoindan can prevent this CPA-induced neurodegeneration of dopaminergic neurons in the absence of functional A1Rs. Since adenosine elevation is widely known to occur in the ageing brain and adenosine and CPA were shown to bind to α-syn N-terminus and cause aggregation, there is a possibility that the increased brain adenosine may be a risk factor for increased α-syn misfolding observed in α-synucleinopathy in PD patients. The results also suggest that targeting α-syn with adenosine-related compounds (e.g., DPCPX and caffeine) or compounds with similar binding profiles (e.g., 1-aminoindan, metformin, nicotine) may be an attractive therapeutic approach to reducing neurodegeneration associated with increased accumulation of adenosine in aging-related neurodegenerative diseases.

## Conclusion

Nanopore analysis and molecular docking techniques are excellent complimentary tools to probe potential protein-drug complexes. Importantly, the combination of nanopore analysis and molecular docking with our in vivo rodent model of α-synucleinopathy in the present study has provided novel insights into the structure and misfolding pattern of the inherently disordered α-syn protein in the presence of adenosine A1R ligands. Here, we demonstrated that adenosine and the A1R agonist CPA bind to the N-terminus of α-syn, similar to 2-aminoindan, thereby promoting a more compact, neurotoxic knot conformation that leads to increased α-syn aggregation and neurodegeneration in the SN and hippocampus (Fig. 5). In contrast, the A1R antagonist DPCPX binds to both the N- and C-termini of α-syn, similar to 1-aminoindan, causing the protein to adopt a neuroprotective loop conformation and thereby reducing neurodegeneration under persistent A1R stimulation. Still, the underlying mechanisms of CPA-mediated α-syn accumulation and aggregation and subsequent neurodegeneration require further studies. Our recent study demonstrated that chronic A1R stimulation with CPA leads to A1R-dependent accumulation of α-syn [25], but other plausible explanation for its intracellular accumulation may also involve downstream A1R signaling, reduced vesicular trafficking of α-syn to the surface membranes, or increased protein stability and reduced degradation of α-syn upon direct binding with A1R ligands. Therefore, stable inhibition of chronic adenosine A1R stimulation occurring in aged brains of PD patients by clinically approved drugs that also promote a “loop” conformation of α-syn may be beneficial neuroprotective therapies to decrease α-synucleinopathy in PD.

## Supplementary Information

**Additional file 1:** **Appendix 1**: Summary of the eight α-synuclein structures as determined by discrete molecular dynamics simulations and further confirmed by far-UV circular dichroism and cross-linking mass spectrometry [47]. **Fig. S1**: Nanopore analysis recordings of α-synuclein alone and with 10 μM CPA and DPCPX (dissolved in methanol). **Fig. S2**: Molecular docking simulation of α-synuclein structures C1 and C3 bound to DPCPX. **Fig. S3**: Molecular docking simulation of α-synuclein C5 structure bound to the five ribonucleosides. **Fig. S4**: Summary of the surface area analysis of the CA1 region of the hippocampus of DAPI, α-synuclein and Thio-S. **Table S1**: Populations and the blockade times of each of translocations and bumping events for α-synuclein alone and α-synuclein complexes with 1% and 10% methanol.

## Data Availability

All the data generated or analyzed during this study are included in this manuscript. Original raw data are available from the University of Saskatchewan (Department of Surgery) and can be readily furnished upon request.

## Abbreviations

DPCPX: 8-Cyclopentyl-1,3-dipropylxanthine
A1R: Adenosine A1 receptor
α-syn: Alpha-synuclein
CPA: N^6^-cyclopentyladenosine
FJC: Fluoro-Jade C
TH: Tyrosine hydroxylase
