# Supplementary material for: Adenosine A1 receptor ligands bind to α-synuclein: implications for α-synuclein misfolding and α-synucleinopathy in Parkinson’s disease
Source: Transl Neurodegener. 2022 Feb 10;11:9. doi: 10.1186/s40035-022-00284-3 (PMC8830172; doi:10.1186/s40035-022-00284-3)
Supplement: Supplementary file 1 — Additional file 1: Appendix 1: Summary of the eight α-synuclein structures as determined by discrete molecular dynamics simulations and further confirmed by far-UV circular dichroism and cross-linking mass spectrometry [47]. Fig. S1: Nanopore analysis recordings of α-synuclein alone and with 10 μM CPA and DPCPX (dissolved in methanol). Fig. S2: Molecular docking simulation of α-synuclein structures C1 and C3 bound to DPCPX. Fig. S3: Molecular docking simulation of α-synuclein C5 structure bound to the five ribonucleosides. Fig. S4: Summary of the surface area analysis of the CA1 region of the hippocampus of DAPI, α-synuclein and Thio-S. Table S1: Populations and the blockade times of each of translocations and bumping events for α-synuclein alone and α-synuclein complexes with 1% and 10% methanol. [file 40035_2022_284_MOESM1_ESM.docx]

**Supplementary Materials and Figure Legends**

Screenshots and sample recordings were obtained to elucidate the behaviour of α-syn in the α-HML pore alone and when it binds to different compounds like CPA and DPCPX (see Supplementary Fig. S1). α-HML is a self-assembling pore and inserts freely in the bilayer membrane. As the pore assembles during a recording, we can observe several pores at once translocating the protein or the protein-drug complex through it.

In addition, two more structures of α-syn (C1 and C3) were studied regarding the binding properties of DPCPX (see Supplementary Fig. S2). As seen in Supplementary Figure 2a, DPCPX forms hydrophobic bonds with the NAC region (amino acids V82, isoleucine 88 (I88), A89 and A90) and the C-terminus (amino acids Y133, D135, and Y136) of the C1 structure of α-syn. DPCPX also forms one hydrogen bond with E137 in the C-terminus of the C1 structure. Lastly, DPCPX binds to the N-terminus and NAC region of the C3 structure. DPCPX forms hydrogen bonds with the positively charged cleft containing K12 and histidine 50 (H50) in the N-terminus of C3. Additionally, it forms hydrophobic bonds with several amino acids in the N-terminus (leucine 8 (L8), A29, L38, threonine 44 (T44), K45, V48, and V55), and in the NAC region (isoleucine 88 (I88) and A89). These results agree with the nanopore analysis results showing that DPCPX binds to α-syn N-terminus, α-syn lacking the NAC domain (∆NAC), and α-syn C-terminus (Fig. 4).


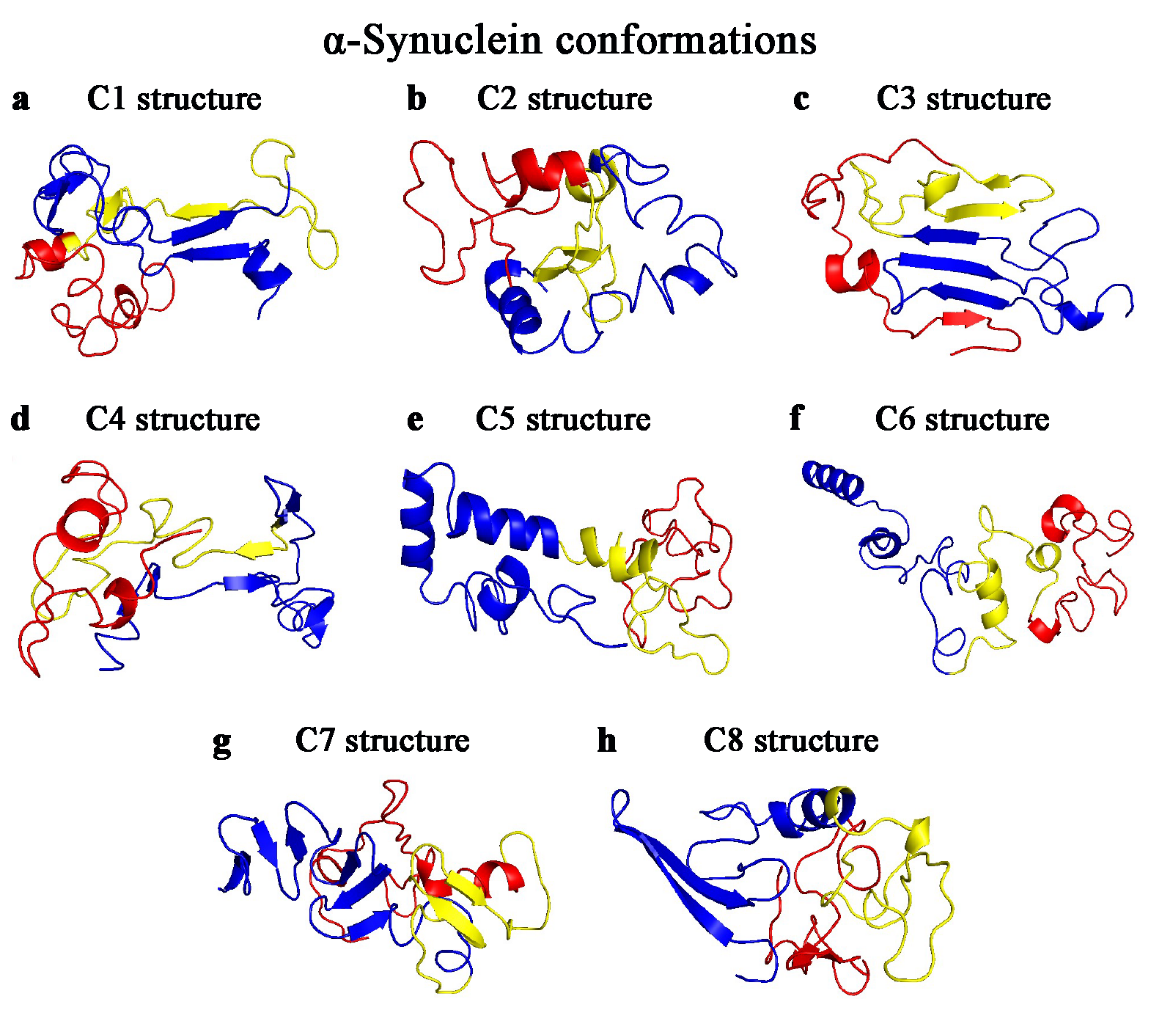


Appendix 1. Summary of the eight α-synuclein structures as determined by discrete molecular dynamics simulations and further confirmed by far-UV circular dichroism and cross-linking mass spectrometry (Chen J, et al. Structure. 2021;29(9):1048-1064.). The α-synuclein domains are denoted as follows: Blue – N-terminus; Yellow – NAC region; Red – C-terminus.


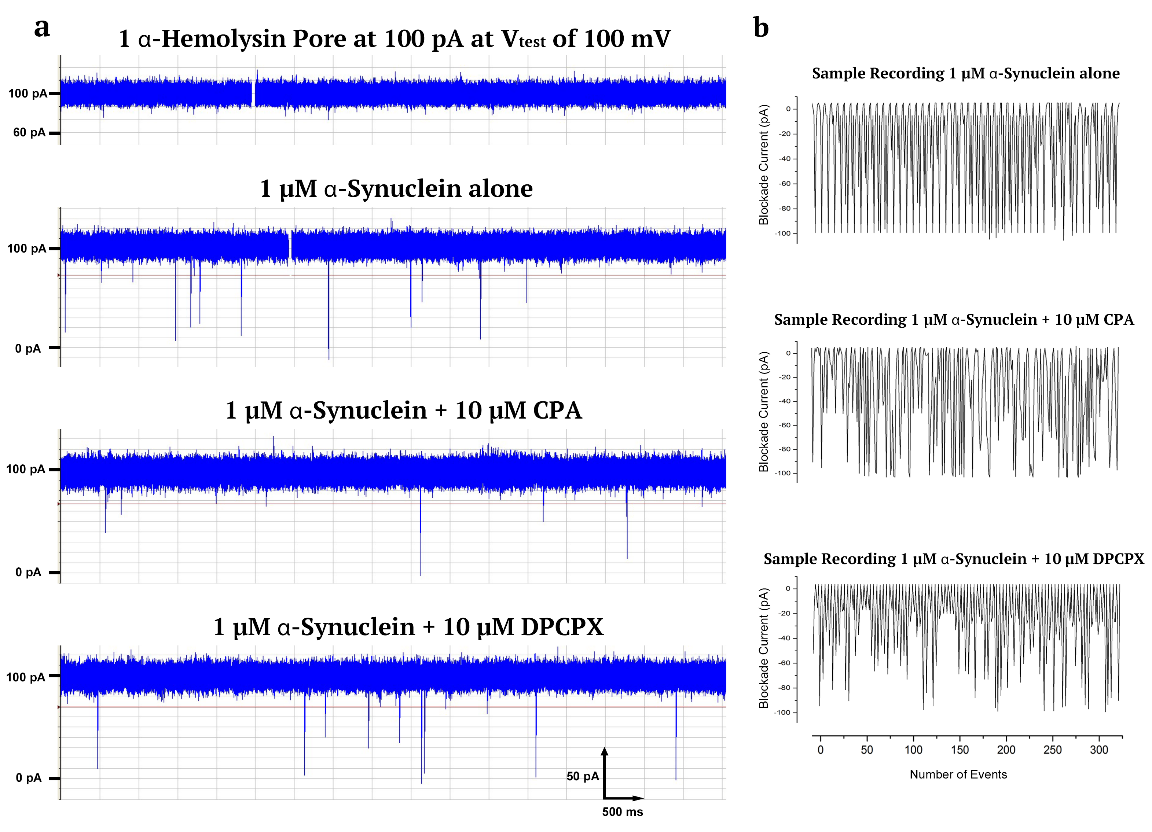


**Figure S1.** Nanopore analysis recordings of α-syn alone and with 10 μM CPA and DPCPX (dissolved in methanol). Patch Clamp recordings of a. Sample baseline current recordings through 1 α-Hemolysin pore when a +100 mV transmembrane potential is applied across the *cis*-side and *trans*-side of the recording chamber. In the presence of α-syn alone, we observe the so-called blockade currents (both translocation and bumping currents) which are altered in the presence of CPA and DPCPX. In particular, the translocation blockade currents (population and current amplitudes) are reduced in the presence of either CPA or DPCPX, whereas intermediate blockade currents (e.g., bumping currents) are increased in population events. b. Blockade current population events (325 representative events per recording) showing a reduction of translocation events and subsequent increase of bumping events when 10 μM CPA or DPCPX is added to α-syn.


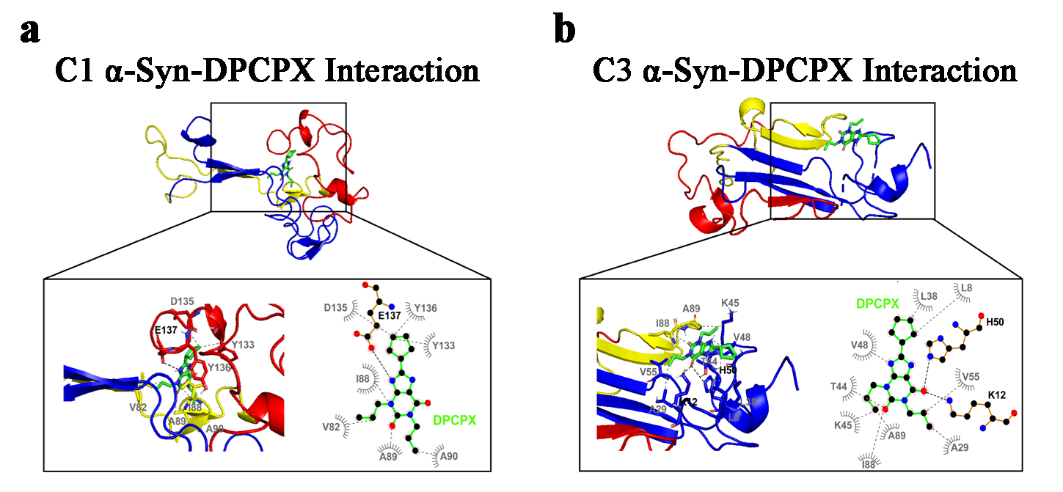


**Figure S2.** Molecular docking simulation of α-synuclein structures C1 (**a**), and C3 (**b**) bound to DPCPX. Below the full 3D representations are shown the magnified binding pocket of α-synuclein and the amino acid residue locations responsible for each drug binding. Bold black dashed lines and amino acid residues indicate hydrogen bonding whereas the grey dashed lines and amino acid residues indicate hydrophobic interactions. DPCPX interacted with the C-terminus and the NAC region (C1 α-syn structure, a.) as well as with the N-terminus and the NAC region (C3 α-syn structure). The molecular docking study was carried out using Autodock Vina module implemented in PyRx tool. Protein and ligand interactions were analyzed and visualized through Pymol and LigPlot+.

**
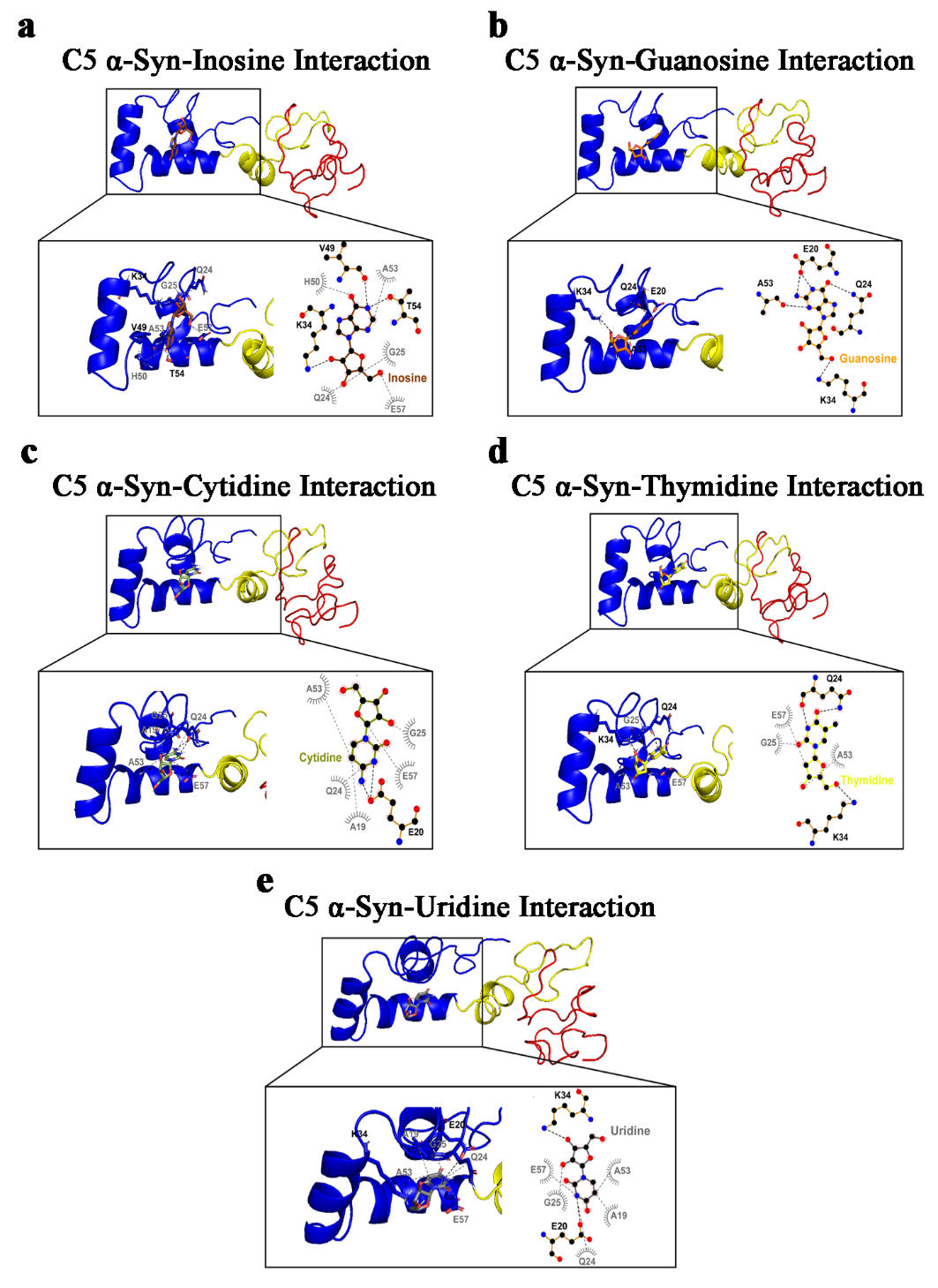
**

**Figure S3.** Molecular docking simulation of α-synuclein C5 structure bound to the five ribonucleosides. In each group the first image is the full 3D representation of the docking of α-synuclein (blue is the N-terminus, yellow the NAC component and red the C-terminus) bound to the two purines: (**a)** Inosine, and (**b)** Guanosine; and the three pyrimidines: (**c)** Cytidine, (**d)** Thymidine, and (**e)** Uridine. Below the full 3D representations are shown the magnified binding pocket of α-synuclein and the amino acid residue locations responsible for each drug binding. The purine guanosine (b) resembles the binding of adenosine to C5 α-syn structure (see Fig. 8b) by forming hydrogen bonds to E20, Q24 and A53 in the N-terminus. In addition, guanosine forms an additional hydrogen bond with K34. The adenosine metabolite inosine forms hydrogen bonds with K34 and two other N-terminal amino acid residues (V49 and T54) located inside the C5 α-syn N-terminus. The pyrimidines cytidine and uridine, like the purines adenosine and guanosine, both form hydrogen bond with E20. Similar to guanosine, the pyrimidines thymidine and uridine form hydrogen bonds with K34. Bold black dashed lines and amino acids indicate hydrogen bonding whereas the grey dashed lines and amino acids indicate hydrophobic interactions. The molecular docking study was carried out using Autodock Vina module implemented in PyRx tool. Protein and ligand interactions were analyzed and visualized through Pymol and LigPlot+.

**
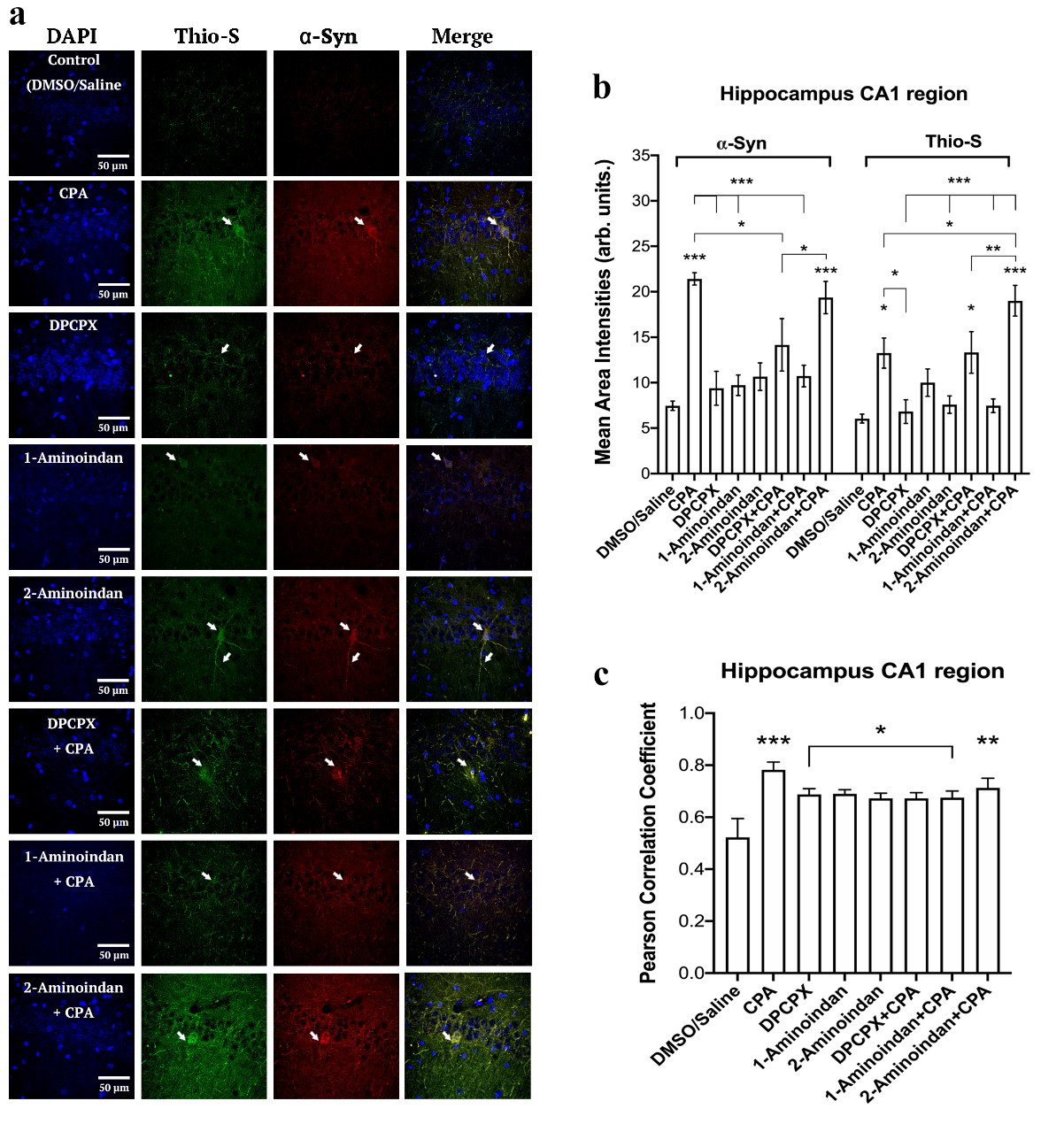
**

**Figure S4.** Summary of the surface area analysis of the CA1 region of the hippocampus of DAPI, α-syn and ThioS. (**a**) Representative images from 40 μm hippocampal rat brain slices after probing for DAPI, anti-α-synuclein and Thioflavin S taken at 63 times magnification with a confocal microscope for the following treatments: Control (DMSO/Saline), CPA, DPCPX, 1-aminoindan, 2-aminoindan, DPCPX + CPA, 1-aminoindan + CPA, and 2-aminoindan + CPA. Scale 50 μm. (**b**) Bar charts showing the mean area intensities of α-syn and Thioflavin S in the CA1 region of the hippocampus of the eight treatments. CPA significantly increased α-syn levels, which was attenuated by DPCPX and 1-aminoindan but not by 2-aminoindan. In contrast, α-syn aggregation (Thio-S staining) was increased by CPA, and this CPA-induced aggregation was attenuated by 1-aminoindan and further enhanced by 2-aminoindan. Fluorescence intensities from a 100 x 100 μm^2^ region from the CA1 pyramidal cell layer of the hippocampus were quantified using a similar method employed for pars compacta region (see Fig. 11). (**c**) Bar chart showing the Pearson correlation coefficient of α-syn and Thioflavin S in the CA1 region of the hippocampus of the eight treatments with CPA. All treatments showed significant colocalization of Thio-S and α-syn signals. Average intensity values and correlation coefficients in bars represent the mean ± SEM from 4 independent experiments. ns, non-significant; **P* < 0.05; ***P* < 0.01; and ****P* < 0.001 (one-way ANOVA followed by Student-Newman-Keuls post-hoc multiple comparison test).

**Table S1.** Populations and the blockade times of each of translocations and bumping events for α-Syn alone and α-Syn complexes with 1% and 10% Methanol.

| Protein - Drug complex | α-Syn | α-Syn +  1% Methanol | α-Syn +  10% Methanol |
| --- | --- | --- | --- |
| Population of  Translocation | 66% | 73% [ns] | 70% [ns] |
| SEM | 1% | 3% | 2% |
| Population of Bumping | 24% | 11% [**] | 30% [ns] |
| SEM | 1% | 1% | 2% |
| Time of Translocation | 0.52 ms | 0.51 ms [ns] | 0.54 ms [ns] |
| SEM | 0.05 ms | 0.05 ms | 0.05 ms |
| Time of Bumping | 0.05 ms | 0.03 ms [ns] | 0.05 ms [ns] |
| SEM | < 0.01 ms | 0.01 ms | < 0.01 ms |

The standard error of the mean (SEM) is calculated in percentage of events for populations and in milliseconds for blockade times. Significances are denoted as: ns p > 0.05; * p < 0.05; ** p < 0.01; and *** p < 0.001 (one-way ANOVA, followed by Student-Newman-Keuls multiple comparison test with the event population or time of α-Syn alone).
